# Chinese medicine, Qijudihuang pill, mediates cholesterol metabolism and regulates gut microbiota in high-fat diet-fed mice, implications for age-related macular degeneration

**DOI:** 10.3389/fimmu.2023.1274401

**Published:** 2023-10-12

**Authors:** Yanqun Cao, Khalid S. Ibrahim, Xing Li, Aileen Wong, Yi Wu, Xu-Dong Yu, Xinzhi Zhou, Zhoujin Tan, Zhiming He, John A. Craft, Xinhua Shu

**Affiliations:** ^1^ Pu Ai Medical School, Shaoyang University, Shaoyang, Hunan, China; ^2^ Department of Biological and Biomedical Sciences , Glasgow Caledonian University, Glasgow, United Kingdom; ^3^ Department of Biology, Faculty of Science, University of Zakho, Zakho, Iraq; ^4^ School of Traditional Chinese Medicine, Hunan University of Chinese Medicine, Changsha, Hunan, China; ^5^ Department of Vision Science , Glasgow Caledonian University, Glasgow, United Kingdom

**Keywords:** traditional Chinese medicine, Qijudihuang pill, age-related macular degeneration, cholesterol, oxidative stress, inflammation, gut microbiota

## Abstract

**Background:**

Traditional Chinese Medicines have been used for thousands of years but without any sound empirical basis. One such preparation is the Qijudihuang pill (QP), a mixture of eight herbs, that has been used in China for the treatment of various conditions including age-related macular degeneration (AMD), the most common cause of blindness in the aged population. In order to explain the mechanism behind the effect of QP, we used an AMD model of high-fat diet (HFD) fed mice to investigate cholesterol homeostasis, oxidative stress, inflammation and gut microbiota.

**Methods:**

Mice were randomly divided into three groups, one group was fed with control diet (CD), the other two groups were fed with high-fat-diet (HFD). One HFD group was treated with QP, both CD and the other HFD groups were treated with vehicles. Tissue samples were collected after the treatment. Cholesterol levels in retina, retinal pigment epithelium (RPE), liver and serum were determined using a commercial kit. The expression of enzymes involved in cholesterol metabolism, inflammation and oxidative stress was measured with qRT-PCR. Gut microbiota was analyzed using 16S rRNA sequencing.

**Results:**

In the majority of the lipid determinations, analytes were elevated by HFD but this was reversed by QP. Cholesterol metabolism including the enzymes of bile acid (BA) formation was suppressed by HFD but again this was reversed by QP. BAs play a major role in signaling between host and microbiome and this is disrupted by HFD resulting in major changes in the composition of colonic bacterial communities. Associated with these changes are predictions of the metabolic pathway complexity and abundance of individual pathways. These concerned substrate breakdowns, energy production and the biosynthesis of pro-inflammatory factors but were changed back to control characteristics by QP.

**Conclusion:**

We propose that the ability of QP to reverse these HFD-induced effects is related to mechanisms acting to lower cholesterol level, oxidative stress and inflammation, and to modulate gut microbiota.

## Graphical Abstract

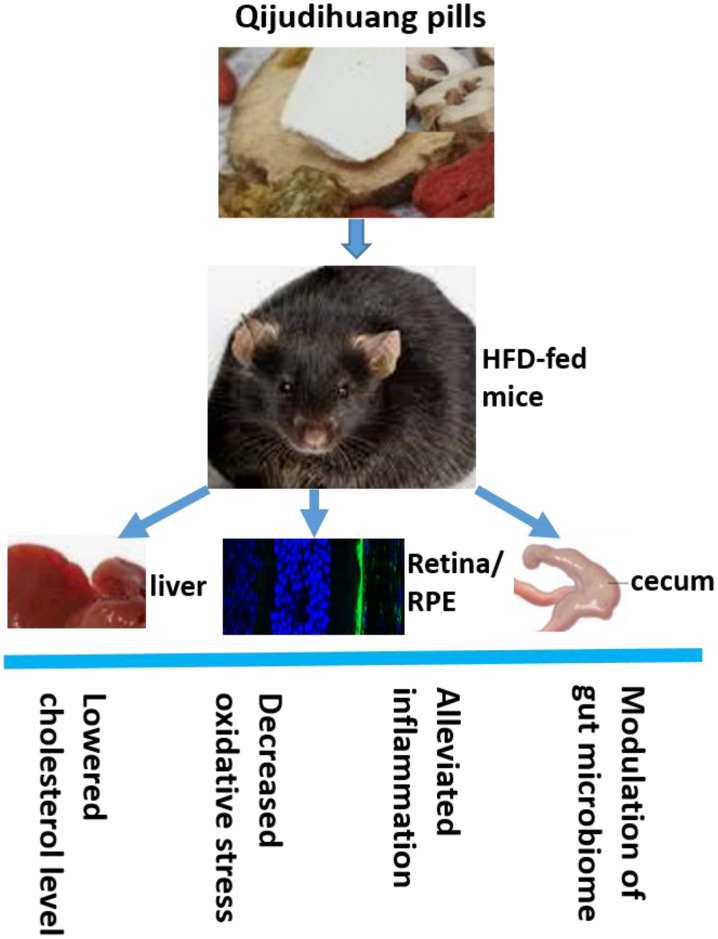

## 1 Introduction

Age-related macular degeneration (AMD) is an incurable visual disorder, which is the third highest cause of blindness in developed countries, with only cataracts and glaucoma as more prevalent eye diseases. Approximately 200 million individuals aged over 50 years are estimated to be affected (1). AMD is chronic in nature and worsens as it progresses from the early and intermediate stages to the late stage. Late AMD is subdivided into dry and wet forms. The dry form is most common and is characterized by the presence of abnormal deposits (drusen) underneath the retinal pigment epithelial (RPE) layer and RPE atrophy. Wet AMD is usually the cause of severe symptoms, such as vision loss, and can cause symptoms to develop in a much shorter timeframe, normally weeks to months. Wet AMD is defined by chronic neovascularization, when new blood vessels, induced by vascular endothelial growth factor (VEGF), grow under the RPE layer and break into the Bruch’s membrane, resulting in bleeding and sudden loss of vision (2). There is no effective treatment for dry AMD, though antioxidants have shown subtle benefits (3).In contrast, anti-VEGF therapy is effective for wet AMD; however, many patients experience incomplete responses, including persistent exudation, hemorrhage, and ongoing lesion fibrosis (4). AMD is a complex disease, associated with environmental and genetic risk factors, and multiple signaling pathways are involved (5).

Cholesterol plays a critical role in the maintenance of cellular structure and function and is associated with various disorders, including AMD (6). Cholesterol has been reported to enrich drusen and form sub-RPE crystals in patients with wet AMD, implicating dysregulation of cholesterol trafficking and metabolism (7, 8). Many studies have demonstrated that cholesterol metabolism and transport genes, apolipoprotein E (APOE), hepatic lipase (LIPC), CETP (cholesteryl ester transfer protein), and ATP Binding Cassette Subfamily A Member 1 (ABCA1) are associated with AMD pathogenesis and progression (9, 10). Knockout of cholesterol efflux-related genes (e.g., *Apoe* and *Abca1*) and cholesterol metabolism genes such as *Cyp27a1* and *Cyp46a1* cause retinal pathology in mice (11–14). We also found that the translocator protein, TSPO, mediated RPE cholesterol efflux, and loss of TSPO caused intracellular accumulation of cholesterol in human and mouse RPE cells (15, 16). High intake of dietary cholesterol has been reported to be associated with an increase in the risk of developing AMD (17–19). A high cholesterol-containing diet causes AMD pathological features in rabbits (20). All these findings suggest cholesterol is involved in AMD pathogenesis and that lowering cholesterol is a therapeutic strategy for patients with AMD.

Traditional Chinese Medicine has been used to treat both dry and wet AMD for thousands of years in China (2). Over 196 prescriptions have been described for treating AMD, among them, Qijudihuang pill (QP), which is regularly prescribed to patients with AMD. QP also shows therapeutic effects in other ocular disorders, such as diabetic retinopathy, retinitis pigmentosa, glaucoma, and dry eye disease (21, 22). QP is made of eight medicinal herbs, namely, *Fructus lycii, Moutan Cortex, Dioscoreae Rhizoma, Corni Fructus, Poria, Alismatis Rhizoma, Flos Chrysanthemi* Indici, and *Rehmanniae Radix Praeparata*. Using a network pharmacology approach, over 134 active compounds have been identified in QP and 72 candidate targets predicted, some of which were reported to have a role in retinal disorders (21). However, the underlying protective mechanisms of QP against ocular diseases, particularly AMD, are not explored.

In the present study, we treated high-fat diet-fed mice with QP and investigated changes in cholesterol metabolism, expression of antioxidant and inflammation genes, and gut microbiome.

## 2 Materials and methods

### 2.1 Preparation of the Chinese medicine QP

For the preparation of QP, 12g of *Fructus lycii*, 10g of *Moutan Cortex*, 15g of *Dioscoreae Rhizoma*, 15g of *Corni Fructus*, 10g of *Poria*, 10g of *Alismatis Rhizoma*, 12g of *Flos Chrysanthemi* Indici, and 30g of *Rehmanniae Radix Praeparata* were pulverized together, put in 75ml distilled water, and boiled for 30 minutes with low heat. The liquid decoction was collected; the remaining material was again put in 75ml water and boiled for 30 minutes with low heat, and the decoction was collected. Both decoctions were mixed and concentrated to reach a final concentration of 1.10g/ml, and this was kept at 4°C.

### 2.2 Animal treatment

All animal work was approved by the local animal welfare committee and followed the UK Home Office Animal Research guidance (Project License PP235815). Four-week-old male C57BL/6J mice were randomly allocated into three groups (eight animals/group). One group was fed with a control diet (CD) and the other two groups were fed for 13 weeks with a high-fat diet (HFD, 78.75% control diet to which 10% lard, 10% corn oil, 1% cholesterol, and 0.25% sodium cholate were added). The body weights of the mice were monitored weekly. After 13 weeks, the CD group and one HFD group were intra-gastrically treated daily with either physiological saline (0.4ml/animal) (CD group) or with the QP decoction (HFD+QP) at a dose of 14.69g/kg, which was calculated based on the clinical application (HFD group). After 30 days of treatment, animals were sacrificed and samples were collected.

### 2.3 Cholesterol measurement

Cholesterol was extracted from untreated, HFD, and HFD QP-treated serum, liver, retina, RPE/choroid with hexane: isopropanol (3:2, v/v). The extract was centrifuged, and the organic fraction was transferred into a new tube and dried under nitrogen gas. The dry lipid was dissolved in a cholesterol-working buffer and the total cholesterol was measured using an Amplex Red Cholesterol Assay kit (Thermo Fisher Scientific, UK) guided by the manufacturer’s protocol.

### 2.4 Quantitative real-time polymerase chain reaction

Total RNA was extracted from untreated, HFD, and HFD QP-treated mouse liver, retina, and RPE/choroid using TRIzol Reagent (Thermo Fisher Scientific, UK) based on the manufacturer’s protocol. CDNA was synthesized and targeted mRNA was detected using kits from Thermo Fisher Scientific, UK following the manufacturer’s instructions. Primers used for qRT-PCR are listed in **Table S1**.

### 2.5 Isolation of bacterial DNA and metagenomic sequencing

Mouse cecum samples were collected and bacterial DNA was extracted using the QIAamp DNA Stool Mini Kit (QIAGEN, UK) based on the manufacturer’s instructions. Purified DNA from individual animals was used for PCR amplification and sequencing of 16S rRNA genes on an Illumina Nova-Seq with 2 x 300 base paired-end reads. Universal primers of the 16S rRNA genes were used to amplify the hypervariable regions, V3-V4 (V3F (338F) and V4R (806R)).

### 2.6 Sequence determination of the gut microbiome based on 16S rRNA amplicons

Sequences were processed with QIIME2 as described previously (23), except for using 230 as the truncation length in DADA2 (24). Reads and metadata have been submitted to the Sequence Read Archive (SRA) with Accession Number PRJNA1005835. The potential, functional metagenome was analyzed with the stand-alone version of PICRUSt2 (25). Raw abundance data was normalized for each pathway to generate Relative Abundance prior to analysis with ANOVA and Tukey’s *post hoc* correction to indicate significance between the three groups. This was conducted in R (v4.2.1) using the rstatix package, and results were plotted with ggplot2. The hierarchical clustering and heatmaps of the predicted proportion MetaCyc pathways and the Kyoto Encyclopedia of Genes and Genomes (KEGG) orthologues (KO) metagenomes were visualized by the SRplot tool (https://www.bioinformatics.com.cn/srplot).

### 2.7 Statistical analysis

Data on body weight, cholesterol level, and gene expression was analyzed using PRISM software (version 9) with one-way or two-way ANOVA followed by an appropriate *post hoc* test. Data was displayed as mean ± SD. 16S rRNA amplicon data was analyzed using Statistical Analysis of Metagenomic Profiles (STAMP) (26) by heatmap to explore the relationship between groups through ANOVA statistical test with Tukey-Kramer *post hoc* test after multiple test corrections. Statistical analysis of the abundance of taxa was conducted with Linear Discriminant Analysis (LDA) Effect Size (LEfSe) (27) using a Galaxy computational tool with settings of LDA at 3. LEfSe determines the features (genes, organisms, clades, and operational taxonomic units) of biologically relevant groupings. Wilcox test of two groups’ comparison (non-parametric) of (Bacteroidetes/Firmicutes ratio) with differences between at least two groups used SRplot online. A *p*-value of < 0.05 was considered significant.

## 3 Results

### 3.1 QP treatment did not affect body weight

During the 13-week initial feeding period, the body weight of the three groups continuously increased. Compared to the control group fed with a normal diet, animals fed with a high-fat diet had a significant increase in body weight from as early as 1 week of feeding that diet (**Figure S1A**). After the subsequent 30-day treatment with QP, the treated animals had similar body weight to the untreated HFD group, suggesting QP did not affect body weight (**Figure S1B**).

### 3.2 QP lowered systemic and local cholesterol level

**Figure 1:**
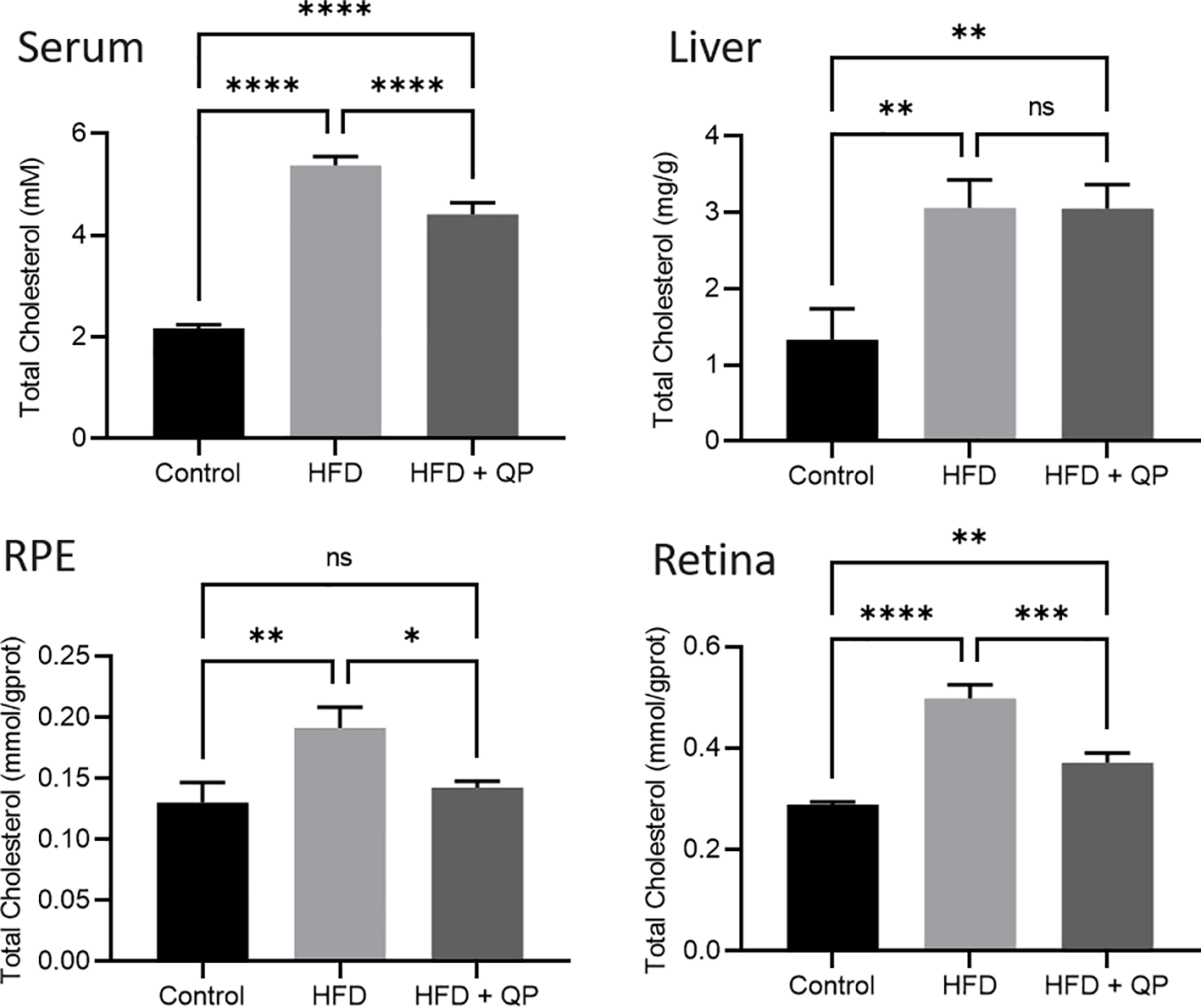
Effect of QP treatment on cholesterol level in serum, liver, RPE, and retina. Data was analyzed using one-way ANOVA followed by Tukey’s multiple comparisons test and presented as mean ± SD. HFD, high-fat diet-fed group; QP, Qijudihuang pill; RPE, retinal pigment epithelial cells. *p<0.05; **p<0.01; ***p<0.001; ****p<0.0001; ns, no significance. Eight samples/group.

**Figure 2:**
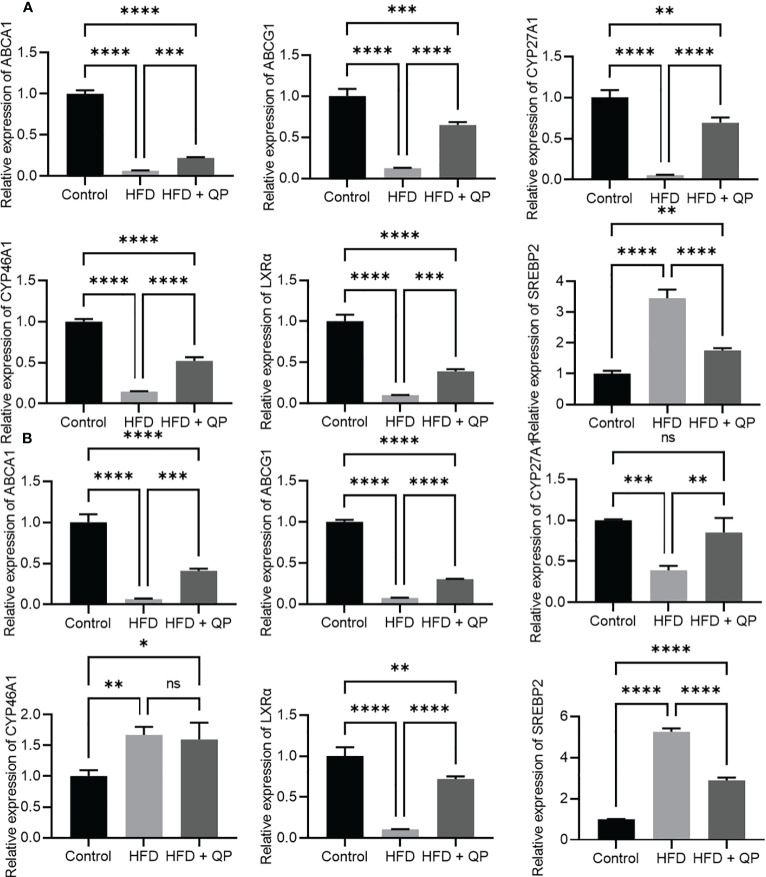
Effect of QP on expression of cholesterol homeostasis genes in RPE/choroid **(A)** and in the retina **(B)**. Data was analyzed using one-way ANOVA followed by Tukey’s multiple comparisons test and presented as mean ± SD. HFD, high-fat diet-fed group; QP, Qijudihuang pill; RPE, retinal pigment epithelial cells. *p<0.05; **p<0.01; ***p<0.001; ****p<0.0001; ns, no significance. Eight samples/group.

High-fat diet causes cholesterol accumulation in tissues and organs of animals and humans (23, 28). In the present study, we further confirmed animals fed with a high-fat diet had a significantly higher level of cholesterol in serum, liver, retina, and RPE/choroid. QP administration reversed the high-fat diet–induced effect on cholesterol levels in serum, liver, retina, and RPE/choroid (**Figure 1**). We also examined the expression of cholesterol homeostasis genes in the liver, RPE/choroid, and retina and found that cholesterol trafficking genes (Abca1 and Abcg1), cholesterol metabolism gene (Cyp27a1), and cholesterol transporting/metabolism-regulating gene (*Nr1h3*, encoding LXRα) had a significant decrease in expression in liver, RPE/choroid, and retina, compared to that of the control animals. QP treatment significantly increased the expression of these genes, compared to that of animals fed with a high-fat diet only. Expression of *Cyp46a1* was markedly downregulated in liver and RPE/choroid of high-fat diet-fed animals, compared to that of control animals, and QP treatment increased its expression, compared to that of animals fed with high-fat diet alone. However, expression of *Cyp46a1* was upregulated in the retina of high-fat diet-fed animals, but not changed in QP-treated animals. The cholesterol synthesis regulator, SCREBP2, had a significant increase in expression in the three tissues of animals fed with a high-fat diet, compared to control animals, whereas QP treatment significantly downregulated the expression of SCREBP2 in the three tissues compared to animals fed with high-fat diets (**Figures 2**, **S2**).

### 3.3 QP modulated expression of antioxidant and inflammation genes

**Figure 3:**
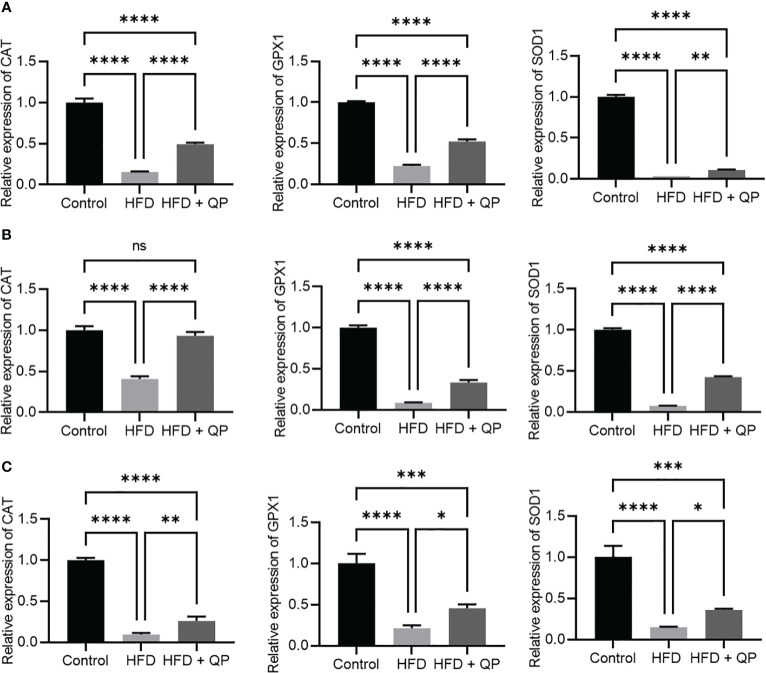
Effect of QP treatment on expression of antioxidant genes in RPE **(A)**, retina **(B)**, and liver **(C)**. Data was analyzed using one-way ANOVA followed by Tukey’s multiple comparisons test and presented as mean ± SD. HFD, high-fat diet-fed group; QP, Qijudihuang pill; RPE, retinal pigment epithelial cells. * p<0.05; ** p<0.01; *** p<0.001; **** p<0.0001; ns, no significance; Eight samples/group, three repeats.

**Figure 4:**
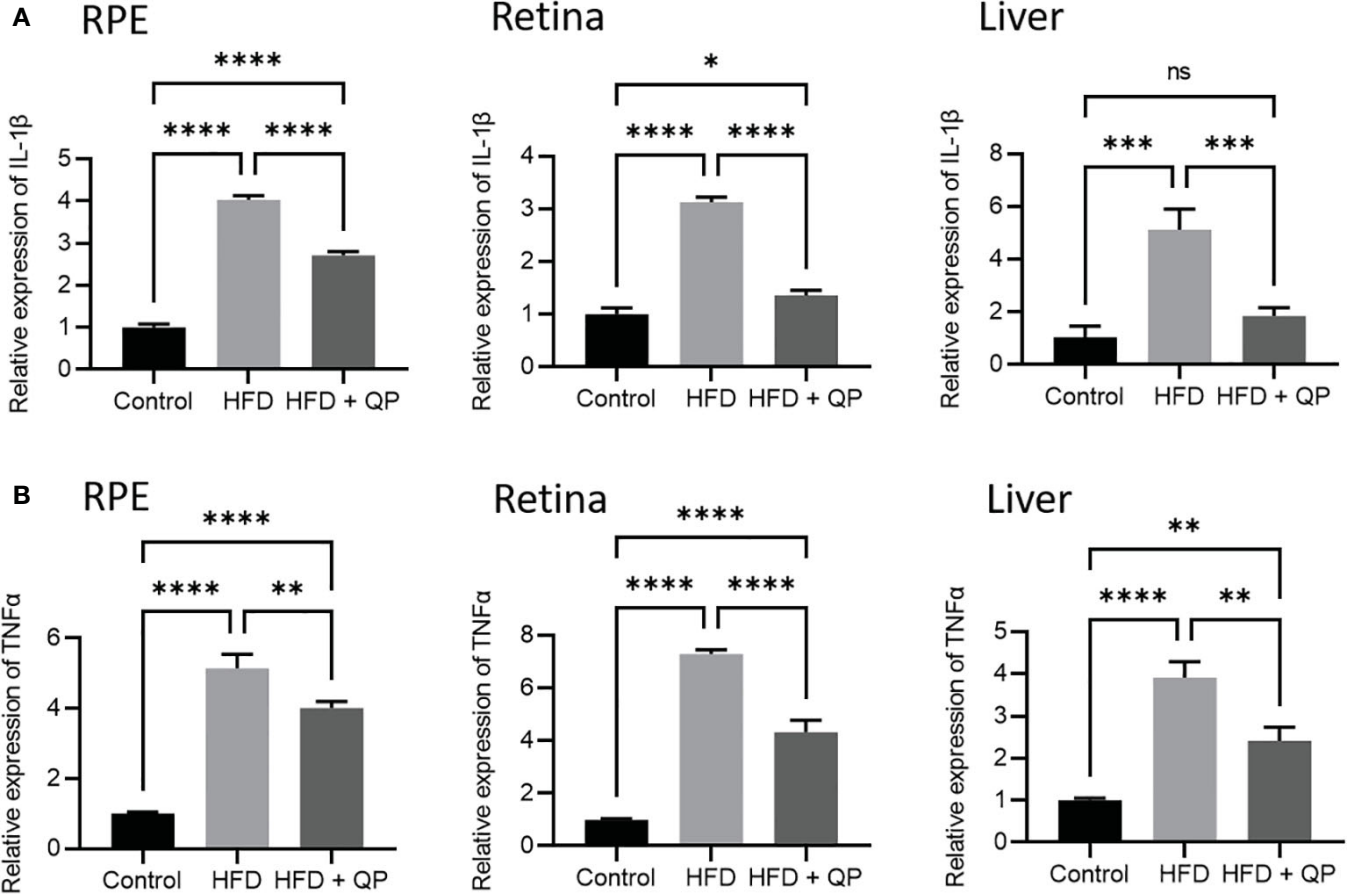
Effect of QP treatment on expression of IL-1β **(A)** and TNFα **(B)** in RPE, retina, and liver. Data was analyzed using one-way ANOVA followed by Tukey’s multiple comparisons test and presented as mean ± SD. HFD, high-fat diet-fed group; QP, Qijudihuang pill; RPE, retinal pigment epithelial cells. * p<0.05; ** p<0.01; *** p<0.001; **** p<0.0001; ns, no significance. Eight samples/group, three repeats.

It is well-documented that a high-fat diet induces oxidative stress and inflammation (29). In the present study, we found that the expression of antioxidant genes (*Catalase*, *Gpx1*, and *Sod1*) was markedly downregulated in RPE/choroid and retina of HFD animals compared to that of CD animals but QP treatment reversed this HFD-induced effect (**Figure 3**). Conversely, expression of pro-inflammatory cytokines (*Il-1β* and *Tnfα*) in RPE/choroid, retina, and liver of HFD mice was markedly upregulated compared to that of CD mice, and QP treatment significantly alleviated expression of the two cytokines in RPE/choroid, retina, and liver in the HFD+QP group (**Figure 4**).

### 3.4 Metagenomic analysis of gut microbiota

**Figure 5:**
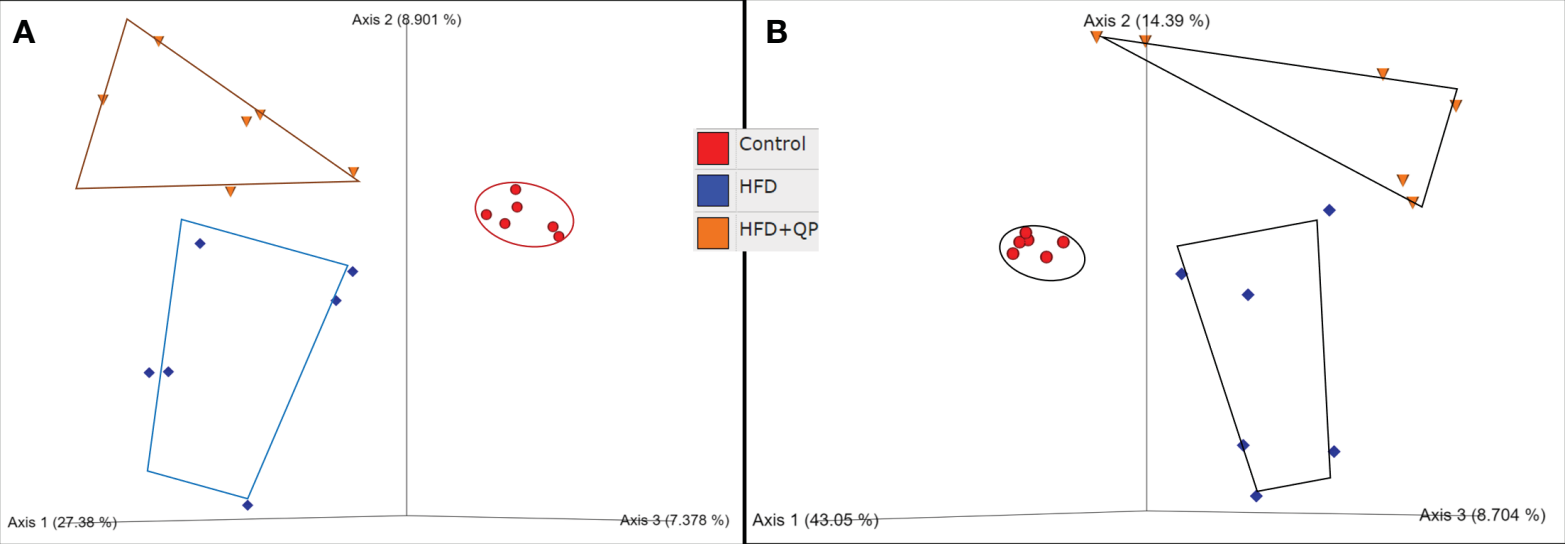
Beta diversity of bacterial communities in the three groups of mice by **(A)** Jaccard and **(B)** Bray-Curtis metrics as shown by Emperor plots. Six samples/group.

Sequence read numbers and the numbers of non-chimeric, joined reads are shown in **Table S2**. Raw read numbers varied between 79700-114311, and after joining and elimination of chimeras, a range of 64.6-91.4% of input survived. Taxonomic profiles of the *organisms* present in each sample were obtained via QIIME2 packages with reference to the Green Genes database at 97% identity (**Figure S3**, **Table S3**). A total of 238 taxa from 8 phyla (Actinobacteria, Bacteroides, Firmicutes, Proteobacteria, Cyanobacteria, Fusobacteria, TM7, and Verrucomicrobia) from 120 genera with 97 identified species and 56 unclassified species are detailed in **Table S3**. Clear differences in bacterial communities between the groups were apparent by diversity analysis (**Figure 5**). Beta diversity indicated a clear separation of microbial communities between CD and HFD as well as between CD and HFD+QP but with some indication of the movement of HFD+QP towards CD. This is further shown in Emperor plots with alternative metrics of determination (**Figure S3**). Additionally, heat maps and hierarchical clustering show a separation between the CD and both high-fat groups (**Figure S4**). The ratio of Bacteroidetes compared to Firmicutes was markedly increased by the HFD compared to control but this ratio was much reduced in the HFD+QP group (**Figure S4C**).

**Figure 6:**
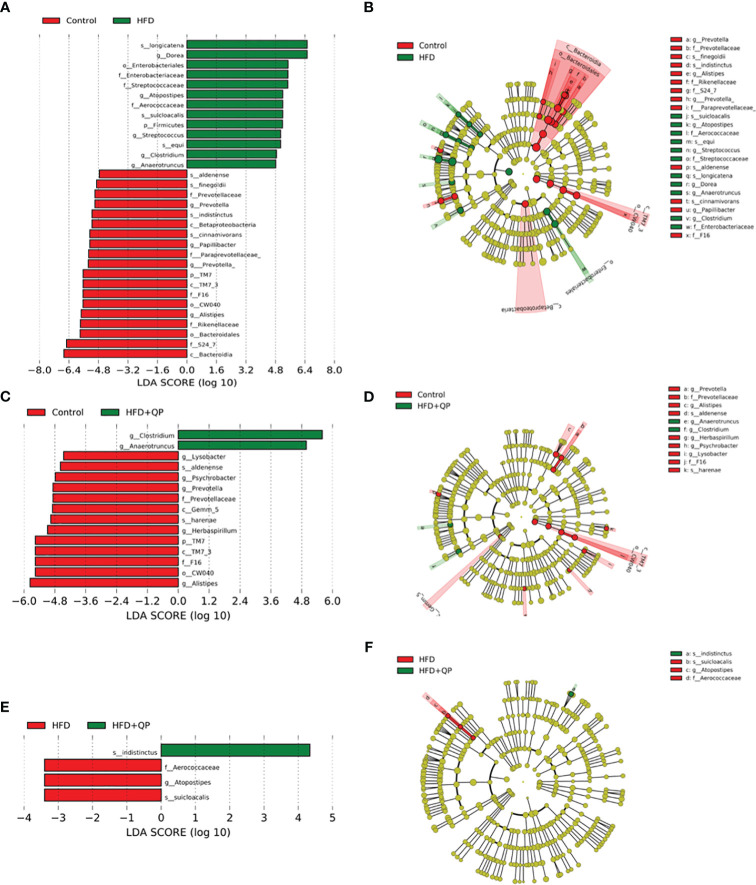
Differential abundance of bacteria in gut microbiome showing pairwise comparison of abundance using the LEfSe method. The figure shows features that are differentially abundant scaled with their effect size **(A, C, E)**, and those differences are mapped to associated taxonomic trees **(B, D, F)**. Effect size is indicated by the LDA score with the green colors showing taxa more abundant in HFD compared to the control group **(A)** and more abundant in HFD+QP compared to control **(C)** and HFD+QP relative to HFD **(F)**. Negative LDA scores are shown in red indicating higher abundance in the comparison partner (**B** Control, **D** Control, and **F** HFD). The parallel, taxonomic representations utilize the same colors: red most abundant, green least abundant, and yellow indicates organisms without significant difference.

The differential abundances of taxa comparing the three experimental groups were determined using the Linear Discriminant Analysis (LDA) Effect Size (LEfSe) and Plot Cladogram (**Figure 6**). Mice fed with HFD showed profound changes in the abundance of taxa compared to taxa in mice given CD. Some taxa were present at significantly higher levels while others were present at much lower levels than found in CD. This is apparent in **Figure 6** where 13 taxa are differentially increased in HFD mice while 19 are decreased. The pattern of changes and identity of taxa are shown in **Figure S5** with the accompanying table. In contrast, HFD+QP had only two taxa with increased abundance (f_Aerococacceae and g_Clostridium) and these were also found when comparing HFD to CD. There was also a smaller list of taxa reduced in HFD+QP (13) of which 8 were common to the other CD comparison while 5 were unique. The complexities of the community changes became more apparent when comparing differential abundance in HFD to HFD+QP. In this case, the number of taxa with increased abundance in HFD was three (each of which was seen in the comparison to CD) and only one with reduced abundance relative to HFD+QP, again a taxa seen in CD (*s_indistinctus*).

### 3.5 Analysis of potential metabolic activities by the gut microbiome

**Figure 7:**
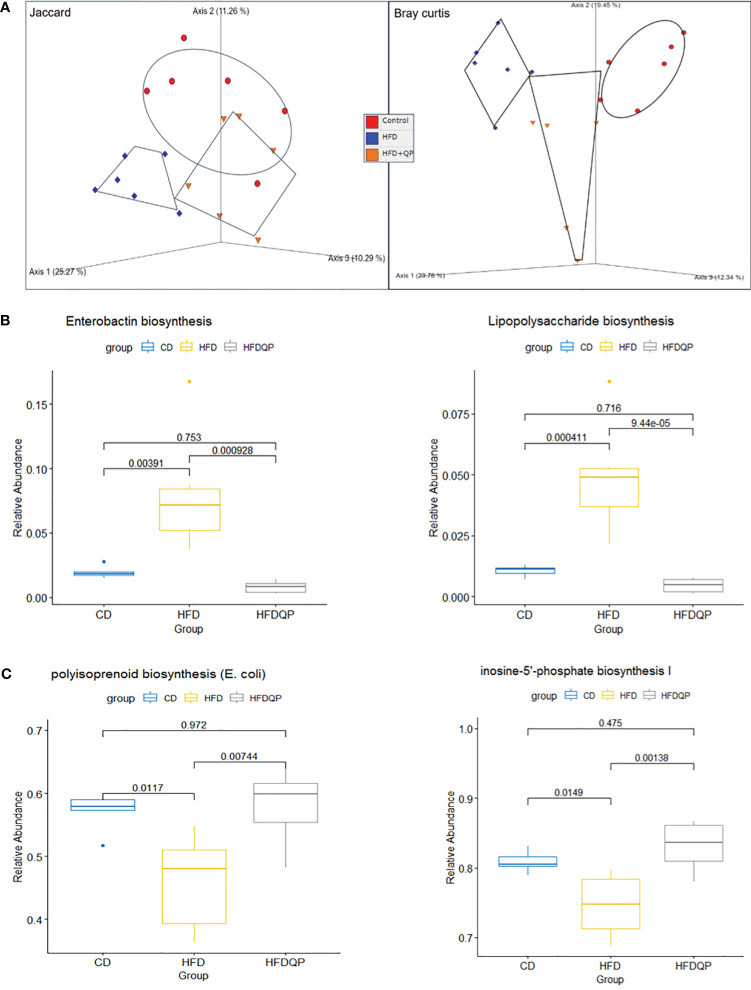
Functional analysis of the metagenome using PICRUSt2 with MetaCyc pathways. Beta diversity of pathways **(A)** by Jaccard and Bray Curtis metrics shown by PCoA. Relative Abundance of selected pathways that promote inflammation **(B)** or pathways of intermediary metabolism **(C)** affected by HFD and reversed by QP. CD, control diet; HFD, high-fat diet; HFDQP, high-fat diet with QP treatment.

We used PICRUSt2 to provide insights into the potential metabolic pathways of the bacterial communities within each of the experimental groups. Analysis with PCoA revealed separation between each of the groups but with a clear movement of the HFD+QP towards CD (**Figure 7A**). Of the 415 MetaCyc pathways, 127 were significantly different between CD and HFD. In 55 of these pathways, there was no significant difference between CD and HFD+QP (**Table S4**). In some pathways (30), HFD increased pathway abundance from CD (**Figure 7B**) and this was reversed back to CD by the QP treatment, and in other cases (9) HFD suppressed abundance and this was increased back to CD by QP (**Figure 7C**).

The pathway changes were further studied by consideration of the Superclass ontologies specified by each of the MetaCyc classifications (**Table S5**). Of the 46 pathways elevated by HFD and reversed by QP, 19 were described by Superclass classification as “Biosynthesis”, 23 as “Degradation/Utilization/Assimilation”, and 4 as “Generation of Precursor Metabolites and Energy”. Of the “Biosynthesis” pathways, nine were involved with “Cofactor, Carrier, and Vitamin Biosynthesis”, mainly with compounds used in Electron Transport Chains, specifically menaquinones. Synthesis of lipopolysaccharides (LPS) and the phenolic siderophore, enterobactin were also notable. In the class “Degradation/Utilization/Assimilation”, “Aromatic Compound Degradation” (five) as well as “C1 Compound Utilization and Assimilation” (three), and “Carboxylic Acid Degradation” (three) along with degradation of amino acids and carbohydrates were represented. Of the other Superclass classifications, all representatives were described by the Superclass term “Generation of Precursor Metabolites and Energy”. Of the nine pathways decreased in HFD but reversed by QP all but one were described by “Biosynthesis”, the other being “Degradation/Utilization/Assimilation”. The representatives in “Biosynthesis” were involved in “Nucleoside and Nucleotide Biosynthesis” (three) or “Sugar Biosynthesis” (two). The example in Degradation was also involved in Purine metabolism.

## 4 Discussion

Although QP has been used to treat AMD in China for a long period, the underlying functional mechanism has not been investigated. In the current study, we treated a high-fat diet-fed mouse model of AMD and found QP decreased HFD-induced elevated levels of cholesterol in the RPE/choroid, retina, liver, and serum, upregulated expression of antioxidant genes, and suppressed expression of pro-inflammatory genes in the RPE/choroid and retinas of high-fat diet-fed animals, as well as modulated gut microbial composition.

Cholesterol accumulates in the drusen of patients with AMD and can be enzymatically or non-enzymatically oxidized into oxysterols (6). Loss of oxysterol-producing enzymes or cholesterol transporters causes retinal degeneration in rodents. HFD exacerbates AMD pathological features in *Apoe* (involved in cholesterol efflux) knockout mice (31). HFD has also been shown to induce oxidative stress, inflammation, and dysbiosis, resulting in accelerated photoreceptor degeneration in rd10 mice (a retinitis pigmentosa mouse model) (32). In the present study, we also demonstrated that feeding with HFD caused increased cholesterol levels, decreased expression of antioxidant genes, and upregulated expression of inflammatory mediators in examined tissues and that these effects were reversed by QP treatment. Network pharmacology predicted that QP contains multiple active compounds and potential protein targets, of which the top ones are involved in inflammation (22). Therefore, it is supposed that there are multiple signaling pathways associated with QP’s protective effect in HFD-fed mice, which requires further investigation.

Excess cholesterol in the retina and RPE is reversibly transported back to the liver and converted to bile acids, principally by mitochondrial Cyp27A1 (33) and Cyp7A1 (34) located in the endoplasmic reticulum. The products are conjugated by taurine or glycine prior to transport into the gall bladder and their release into the duodenum is stimulated by feeding. The majority of the secreted BA is reabsorbed following deconjugation in the ileum to complete the BA enterohepatic circulation, while a small proportion arrives in the colon (35). Gut bacteria metabolize bile acids by a wide variety of reactions including deconjugation, re-conjugation, dihydroxylation, and oxidation (36, 37). The diversity of gut bacteria and their metabolic capacity to act on BAs determines effects on host systems via a multitude of BA-activated signal receptors that regulate hepatic cholesterol metabolism by affecting the expression of enzymes causing their formation, including the two Cyps (37).

When mice are provided with a high-fat diet there are multiple effects on cholesterol metabolism, including conversion to bile acids. These alter the spectrum of bacteria in the gut, and thus the signaling through bile acids, to affect the host including on the gut-brain axis (38). Indeed, HFD causes widespread changes in microbiota communities, leading to dysbiosis associated with obesity, type 2 diabetes, and AMD as well as a number of other health-compromising conditions (39–41). Those effects were observed in this study at the phylum level with the frequently observed increase of the ratio of Bacteroidetes compared to Firmicutes. The changes can also be seen in terms of beta diversity with the PCoA revealing a clear separation between the CD and HFD groups. Furthermore, analysis with the LEfSe tool revealed an HFD-associated increase in abundance of a variety of taxa previously commented on by ourselves and others (23, 42–44). At the same time, other taxa were decreased by this diet. For instance, Anaerotruncus was increased and has been associated with HCC and non-alcoholic steatohepatitis (45), whereas beneficial f-S27-4 was decreased (23, 43).

One objective of this project was to ascertain whether QP has an effect on gut microbiome and thus whether the observed changes play a mechanistic contribution to potential therapeutic effects. Even while the HFD was continued, QP, at least in part, reversed the effects observed with HFD alone. Thus, the spectrum of phyla reverted more towards that seen in CD as illustrated by a decrease of Bacteroidetes/Firmicutes, a movement of the HFD+QP group towards CD in PCoA plots, and a more restricted change in abundances of taxa in LEfSe, both for those that increased and those that decreased in abundance. The number and identity of taxa that were either increased or decreased in HFD+QP vs. CD were significantly diminished compared to when HFD alone was compared to CD.

Changes in the composition of microbial communities will have effects on global metabolic capacity and thus on interaction with the host. We examined the extent and nature of such potential changes with PICRUSt2. On a global scale, using diversity measures, clear changes induced by HFD and partially reversed by QP were apparent in PCoA plots, changes that closely parallel those seen when considering taxa alone. In terms of individual pathways increased by HFD, those generating pro-inflammatory effectors were notable. These included the synthesis of enterobactin, polymixin B, and lipopolysaccharide, each of which was increased by HFD but reversed when QP was included in the diet. We propose that the increase of IL-1B and TNF-a in the liver, RPE/choroid, and retina are also associated with these changes occurring in the gut microbiota due to HFDs. Likewise, we propose that the decrease of anti-oxidative enzymes catalase, GPX, and SOD1 is a response to changes in the abundance of bacterial taxa, their associated metabolic capacity, and nature. For instance, Enterobactin is a siderophore and derivative of 2,3 dihydroxy N-benzoylserine lactone that chelates primarily ferric iron and is produced by Gram-negative bacteria (46). Since iron is a requirement of host and microbes, an imbalance of iron uptake will impair host systems, and enterobactin has been shown to promote bacterial colonization (30) and interfere with host immune responses (47). Polymixins are microbially-produced antibiotics that illustrate two effects of the HFD. The production of polymixin antibiotics in the gut provides one mechanism leading to the remodeling of bacterial communities and indeed the original interest in these compounds arose as they provided a prospect for the treatment of multidrug-resistant organisms (48). Aligning with this mechanism of remodeling are bacteria, such as Pseudomonas, that have resistance to polymixin (49). However, the drugs also produced marked nephrotoxicity, illustrating the second point that the change of bacterial communities results in the formation of toxic compounds, causing harm to the host. The mode of action of polymixins is thought to be by interaction with phospholipids, causing membrane disruption including macrophages, and stimulating the production of IL-1β (50). An alternative theory suggests that polymixins induce oxidative stress caused by the formation of reactive oxygen species (51), which resonates with altered oxidative resistance in RPE. Resistant bacteria occur in Escherichia, Klebsiella, Salmonella, Shigella, Enterobacter, and Citrobacter (48), and have altered LPS structures that block the interaction with antibiotics (52). Other possible contributors to intestinal inflammation are changes to purinogenic pathways as found in inflammatory bowel disease (53). These pathways were found to be lowered by the HFD but raised to control levels when QP was administered.

While the complex effects of HFD on bacteria and their interaction with the host are starting to be understood, QP has not been previously explored at the levels described here. We have started to characterize the component chemicals present in the QP plant extract (in preparation) and established the principle components. How these might interact with the host and bacteria demands future investigation but one possibility is that they act with the promiscuous nuclear hormone receptor pregnane-X receptor (PXR) located in the liver and gut enterocytes. PXR regulates the expression of many Phase 1 and Phase 2 enzymes and thus affects the metabolism of cholesterol to BAs, for instance, the induction of SULT2A, which conjugates BAs prior to export, and of Mrp2 and Oatp2, the export components (54).

TCM preparations have widely shown lipid-lowering function in dyslipidemia (55, 56). For targeting cholesterol, TCM can inhibit intestinal absorption of cholesterol, suppress endogenous cholesterol synthesis, promote cholesterol reverse transport and excretion, and regulate the expression of cholesterol homeostasis-associated transcription factors (55, 56). Berberine, a major functional compound of many medicinal herbs, has been shown to lower blood cholesterol in atherogenic-diet-fed rats at least partly via the inhibition of intestinal cholesterol absorption (57). TCM Jiang-Zhi-Ning, containing four Chinese herbs, can significantly lower cholesterol, triglyceride, and low-density lipoprotein-cholesterol in hyperlipidemic rats, partly by inhibiting the expression of 3-hydroxy-3-methylglutaryl-CoA reductase (HMGCR), the rate-limiting enzyme for cholesterol synthesis (58). TCM FufangZhenzhuTiaoZhi has been shown to decrease plasma cholesterol in hyperlipidemic rats via downregulating HMGCR expression and increasing expression and activity of 7-α hydroxylase (CYP7A1, the rate-limiting enzyme for bile acid synthesis) (59). In the present study, our data suggested that the effect of QP in lowering cholesterol is possibly through the promotion of cholesterol metabolism, transport, and excretion. It will be worth investigating whether QP inhibits cholesterol intestinal absorption and endogenous synthesis. Additionally, there are reports about the hepatotoxicity of TCM and natural products in animal models and human patients (60, 61), so it may be necessary to examine whether there is any potential toxicity of QP in animal models and patients with AMD.

## 5 Conclusion

This study aimed to investigate the relationship between HFD and AMD and start to unravel the mechanisms whereby QP may provide relief from the condition. HFD was shown to exacerbate lipid accumulation in the RPE and retina and associated inflammation and oxidative stress. Changes in the gut microbiota, their metabolic capacity, and signaling between the host and microbes will have contributed to these pathological conditions. We propose that the ability of QP to reverse these effects is related to mechanisms acting to lower cholesterol levels, oxidative stress, and inflammation, and to modulate gut microbiota; and these will be investigated in future studies.

## Data availability statement

The datasets presented in this study can be found in online repositories. The names of the repository/repositories and accession number(s) can be found in the article/**Supplementary Material**.

## Ethics statement

The animal study was approved by The Animal Welfare Committee, Glasgow Caledonian University. The study was conducted in accordance with the local legislation and institutional requirements.

## Author contributions

XS: Conceptualization, Funding acquisition, Writing – original draft, Writing – review & editing. YC: Investigation, Writing – original draft. KSI: Formal Analysis, Writing – original draft. XL: Investigation, Writing – original draft. AW: Investigation, Writing – original draft. YW: Investigation, Writing – original draft. XDY: Investigation, Writing – original draft. XZ: Formal Analysis, Writing – original draft. ZT: Project administration, Writing – original draft. ZH: Project administration, Writing – original draft. JAC: Formal Analysis, Writing – original draft, Writing – review & editing.
