# Supplementary material for: Chinese medicine, Qijudihuang pill, mediates cholesterol metabolism and regulates gut microbiota in high-fat diet-fed mice, implications for age-related macular degeneration
Source: Front Immunol. 2023 Oct 12;14:1274401. doi: 10.3389/fimmu.2023.1274401 (PMC10602650; doi:10.3389/fimmu.2023.1274401)
Supplement: Supplementary file 1 [file DataSheet_1.docx]

Supplementary Data


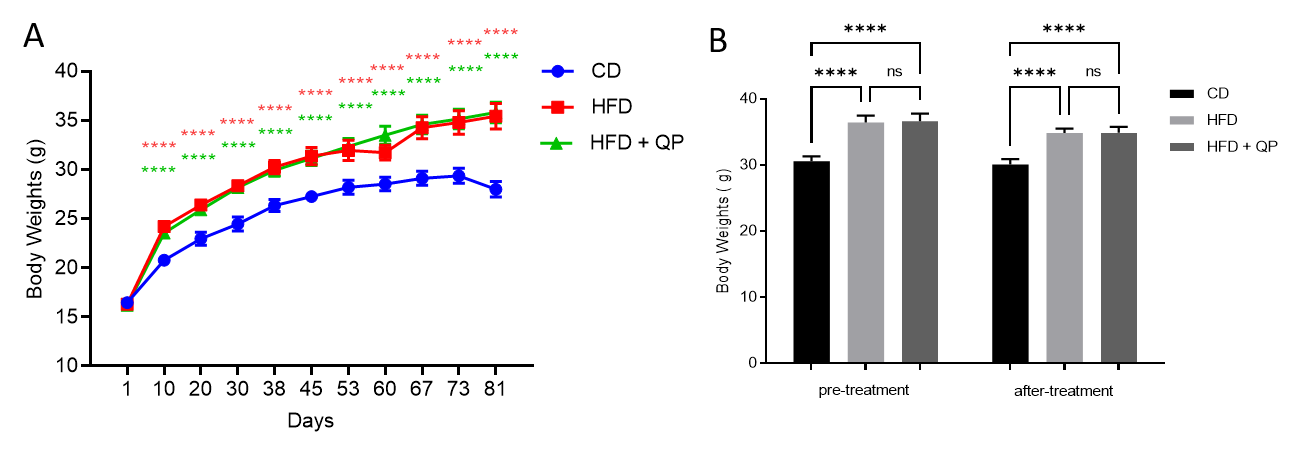


**Figure S1** (A) Increased bodyweight in high-fat diet fed mice. Data was analyzed using two-way ANOVA followed by Dunnett’s multiple comparisons test and presented as mean ± SD. (B) QP treatment had no effect on bodyweight in treated mice. Data was analyzed using two-way ANOVA followed by Tukey’s multiple comparisons test and presented as mean ± SD. CD, control diet; HFD, high-fat diet group; HFD+QP, high-fat diet fed mice treated with QP (qijudihuang pill). **** p<0.00001; ns, no significance. 8 animals/group.

**
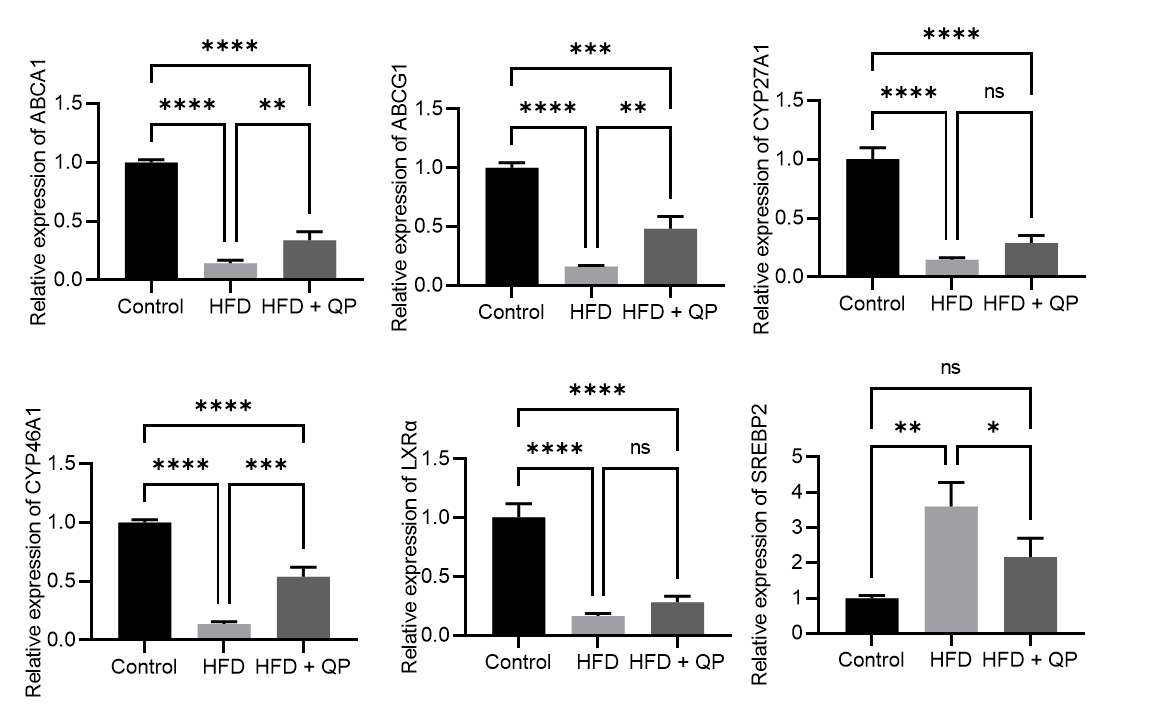
**

**Figure S2** Effect of QP treatment of expression of cholesterol homeostasis genes in liver. Data was analyzed using one-way ANOVA followed by Tukey’s multiple comparisons test and presented as mean±SD. HFD, high-fat diet fed group; QP, Qijudihuang pill; RPE, retinal pigment epithelial cells. * p<0.05; ** p<0.01; *** p<0.001; **** p<0.0001; ns, no significance. 8 samples/group.


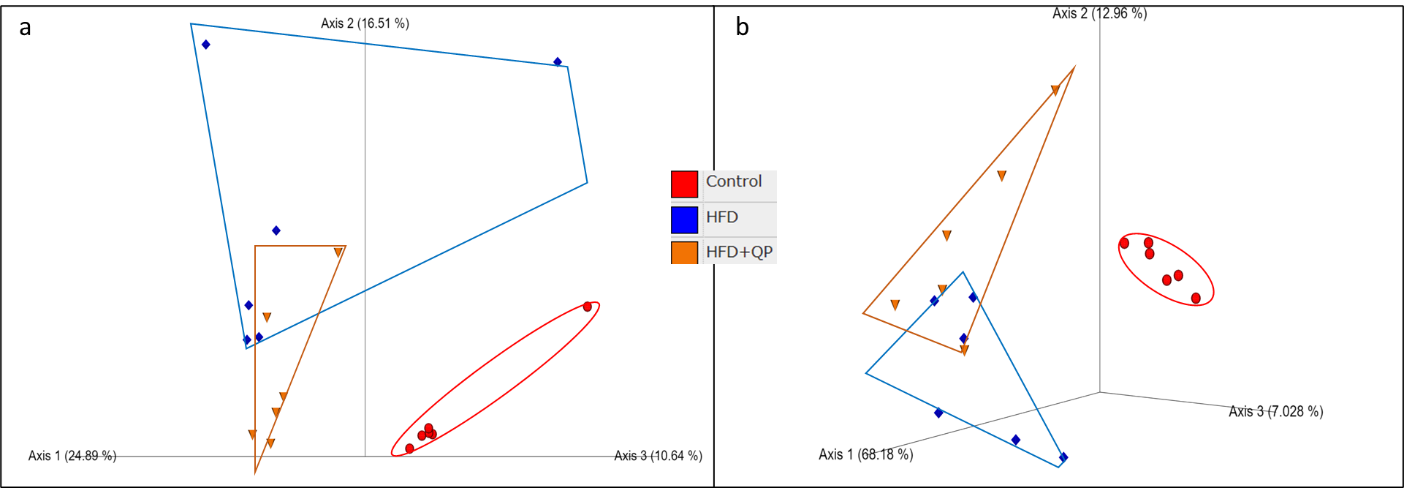


**Figure S3** Beta diversity among three experimental groups of mice. PCoA of taxa based on unweighted_unifrac (a) and weighted_unifrac_approaches (b). Keys; Control= normal diet mice group, HFD = High fat diet mice group, HFD+QP = High fat diet + treat with QP mice group. 6 samples/group.


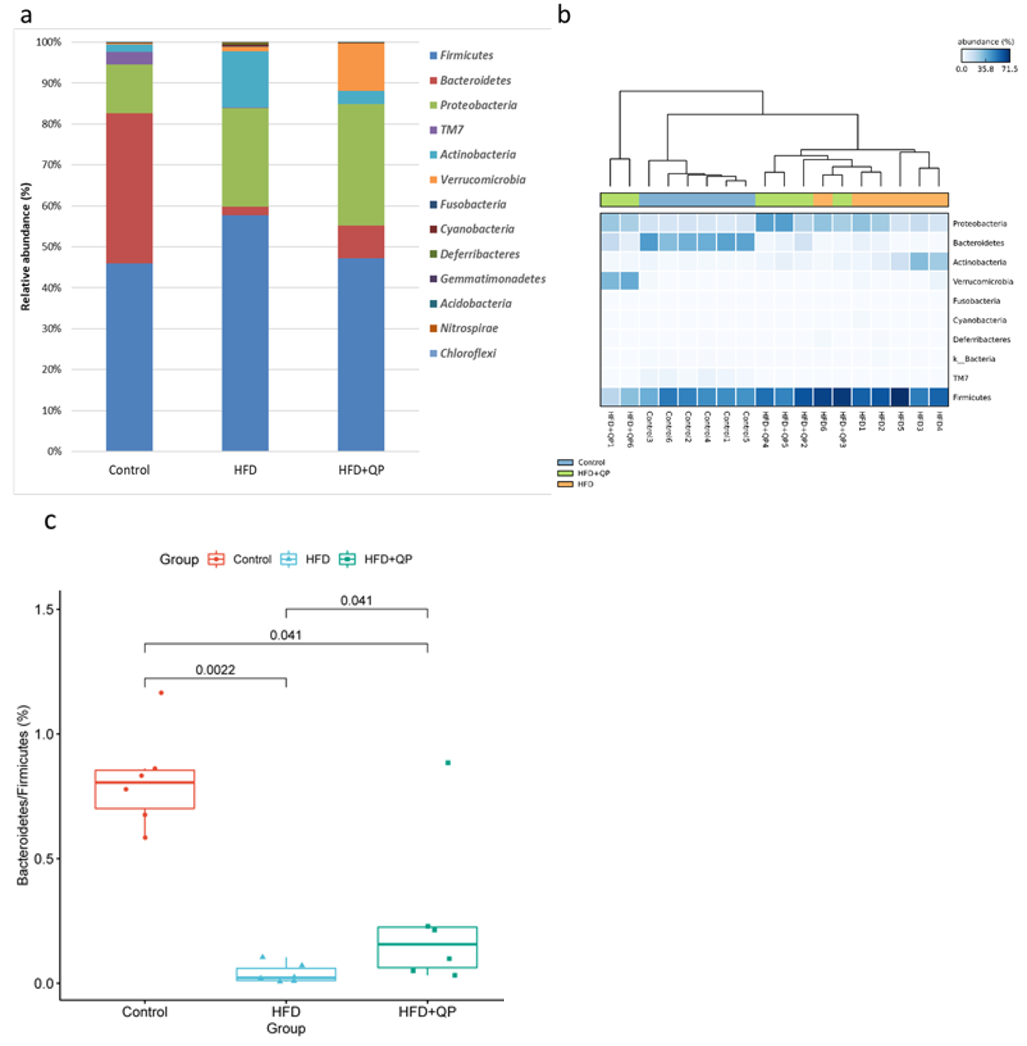


**Figure S4** Relative abundance among three experimental groups of gut phyla (a) and a corresponding heatmap (b) and a comparison between the groups of the ratios of abundance of phyla Bacteroidetes and Firmicutes (c). 6 samples/group.

**
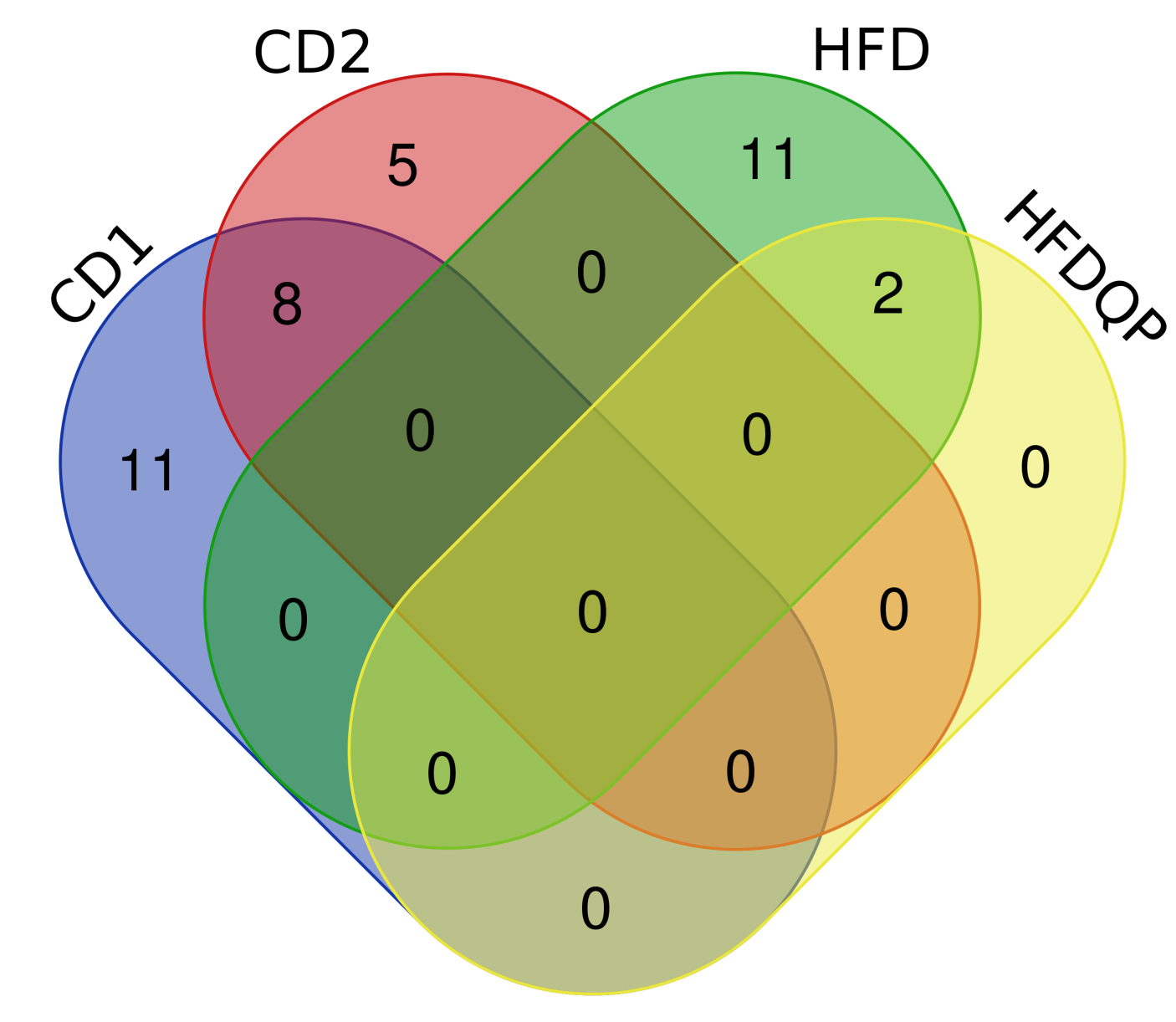
**

**Figure S5** Analysis of taxa abundance complexity between the three groups. A comparison was made of the taxa identified as altered in abundance in HFD relative to CD (CD1) and in HFD after QP treatment relative to CD (CD2). The table shows shared and unique taxa (6 samples/group).

| Names | total | taxa |
| --- | --- | --- |
| HFD HFD+QP | 2 | f_Aerococacceae,g_Clostridium |
| CD1 CD2 | 8 | f_F16,g_Alistipes,o_CWO40,g_Prevotella,s_aldense,p_TM7,c_TM7_3, f_Provetellaceae |
| HFD | 11 | p_Firmicutes,f_Streptococcaceae, s_longicatena, g_Anearotruncus, g_Atopostipes, s_equi,g_Stroptococcus,s_suiclocalis, f_Enterobacteriaceae,g_Dorea, o_Enterobacteriales |
| CD1 | 11 | f_S24_7,o_Bacterodales,s_cinnamvirans, f_Rickenellaceae, g_Prevotells2,s_fingoldii, s_indistinctus,c_Betaproteobacteria, p_Papillobacter,f_Paravotellaceae, c_Bacteroidia, |
| CD2 | 5 | c_Gemm_5,s_hareneae,g_Herbospirillum,g_Lysobacter,g_Psychrobacter |

**Table S1** Primers were used for qRT-PCR

| **Gene** | **Primer Sequences** | |
| --- | --- | --- |
|  | **Forward (5'–3')** | **Reverse (5'–3')** |
| β-actin | ACATCCGTAAAGACCTCTATGCC | TACTCCTGCTTGCTGATCCAC |
| Catalase | GCGGACATTCTACACAAAGGT | CAGTGAAATTCTTGACCGCTT |
| SOD1 | GAACCATCCACTTCGAGCAG | CAACATGCCTCTCTTCATCCG |
| GPX1 | CGGAATGCCTTGCCAACACC | CAAAGTTCCAGGCAATGTCGTT |
| IL-1β | TGAAATGCCACCTTTTGACAGT | TTCTCCACAGCCACAATGAGT |
| TNF-α | AGCACAGAAAGCATGATCCG | CACCCCGAAGTTCAGTAGACA |
| LXRα | TCCTGATTCTGCAACGGAGT | CCCTATCCCTAAAGCAACCCC |
| CYP27A1 | ATCCTACATCCATTCGGCTCT | GTTCTGCAATCCTCCGACCC |
| CYP46A1 | TTGGGGAGAGACTGTTTGGC | TGTCTGTCCATCTGCCTTAGC |
| ABCA1 | CTTGTTCAGTGTTGCCCATGCC | ACAGTCAGCACAAACCAACCCA |
| ABCG1 | GCCGCTCCATCGTCTGTACCAT | GGCAGTTCAGACCCAGATCCCT |
| SREBP2 | AGCAACGGGACCATTCTGAC | ATATTGTGTGTTGTCCGCCTCT |

**Supplementary Table S2** Total counts for raw and trimmed reads (Basic statistics for read counts and data output)

| sample-id | input | filtered | percentage of input passed filter | denoised | merged | percentage of input merged | non-chimeric | percentage of input non-chimeric |
| --- | --- | --- | --- | --- | --- | --- | --- | --- |
| sample1 | 80107 | 79714 | 99.51 | 77715 | 68826 | 85.92 | 51769 | 64.62 |
| sample2 | 80401 | 80099 | 99.62 | 78485 | 71549 | 88.99 | 59262 | 73.71 |
| sample3 | 80372 | 79835 | 99.33 | 78463 | 71514 | 88.98 | 61556 | 76.59 |
| sample4 | 80160 | 79696 | 99.42 | 77811 | 68640 | 85.63 | 51921 | 64.77 |
| sample5 | 79868 | 79469 | 99.5 | 77361 | 69194 | 86.64 | 54011 | 67.63 |
| sample6 | 79796 | 79211 | 99.27 | 77202 | 66995 | 83.96 | 53694 | 67.29 |
| sample7 | 79700 | 79278 | 99.47 | 77203 | 70244 | 88.14 | 62140 | 77.97 |
| sample8 | 114311 | 113607 | 99.38 | 110811 | 101187 | 88.52 | 87486 | 76.53 |
| sample9 | 80277 | 79925 | 99.56 | 78557 | 75890 | 94.54 | 73398 | 91.43 |
| sample10 | 80084 | 79869 | 99.73 | 78595 | 75660 | 94.48 | 70746 | 88.34 |
| sample11 | 79676 | 79404 | 99.66 | 77222 | 70606 | 88.62 | 57663 | 72.37 |
| sample12 | 79990 | 79596 | 99.51 | 78255 | 74910 | 93.65 | 67910 | 84.9 |
| sample13 | 79966 | 79667 | 99.63 | 78484 | 75671 | 94.63 | 71642 | 89.59 |
| sample14 | 79842 | 79437 | 99.49 | 78085 | 74565 | 93.39 | 70045 | 87.73 |
| sample15 | 80113 | 79704 | 99.49 | 78180 | 74590 | 93.11 | 70875 | 88.47 |
| sample16 | 111802 | 111110 | 99.38 | 108846 | 101175 | 90.49 | 88163 | 78.86 |
| sample17 | 79809 | 79458 | 99.56 | 78418 | 74637 | 93.52 | 67040 | 84 |
| sample18 | 79849 | 79491 | 99.55 | 78777 | 75946 | 95.11 | 71241 | 89.22 |

**Supplementary Table S3: Taxonomic profile of each sample**

|  | group | Control | Control | Control | Control | Control | Control | HFD | HFD | HFD | HFD | HFD | HFD | HFD+QP | HFD+QP | HFD+QP | HFD+QP | HFD+QP | HFD+QP |
| --- | --- | --- | --- | --- | --- | --- | --- | --- | --- | --- | --- | --- | --- | --- | --- | --- | --- | --- | --- |
|  | index1 | Control1 | Control2 | Control3 | Control4 | Control5 | Control6 | HFD4 | HFD5 | HFD6 | HFD1 | HFD2 | HFD3 | HFD+QP1 | HFD+QP2 | HFD+QP3 | HFD+QP4 | HFD+QP5 | HFD+QP6 |
|  | index | sample1 | sample2 | sample3 | sample4 | sample5 | sample6 | sample7 | sample8 | sample9 | sample10 | sample11 | sample12 | sample13 | sample14 | sample15 | sample16 | sample17 | sample18 |
| 1 | k__Bacteria;p__Firmicutes;c__Clostridia;o__Clostridiales;f__;g__;s__ | 679 | 283 | 2675 | 3293 | 946 | 991 | 2248 | 2891 | 1086 | 3602 | 7972 | 2821 | 112 | 23638 | 6717 | 20715 | 3885 | 297 |
| 2 | k__Bacteria;p__Bacteroidetes;c__Bacteroidia;o__Bacteroidales;f__[Odoribacteraceae];g__Butyricimonas;s__ | 4 | 9 | 15 | 10 | 0 | 14 | 31 | 17 | 0 | 0 | 0 | 6 | 185 | 112 | 12 | 27 | 10 | 49 |
| 3 | k__Bacteria;p__Actinobacteria;c__MB-A2-108;o__0319-7L14;f__;g__;s__ | 0 | 0 | 0 | 0 | 0 | 0 | 0 | 141 | 0 | 0 | 0 | 0 | 0 | 0 | 0 | 31 | 0 | 0 |
| 4 | k__Bacteria;p__Firmicutes;c__Clostridia;o__Clostridiales;f__Ruminococcaceae;g__Butyricicoccus;s__pullicaecorum | 156 | 494 | 108 | 81 | 204 | 596 | 114 | 101 | 11 | 17 | 45 | 0 | 6 | 319 | 372 | 148 | 117 | 32 |
| 5 | k__Bacteria;p__Bacteroidetes;c__Flavobacteriia;o__Flavobacteriales;f__Flavobacteriaceae;__;__ | 50 | 44 | 31 | 45 | 20 | 25 | 17 | 58 | 20 | 19 | 37 | 27 | 16 | 15 | 10 | 51 | 10 | 11 |
| 6 | k__Bacteria;p__Bacteroidetes;c__Bacteroidia;o__Bacteroidales;f__S24-7;g__;s__ | 11142 | 15086 | 20214 | 11138 | 13000 | 10853 | 294 | 329 | 136 | 23 | 35 | 31 | 121 | 2184 | 155 | 557 | 226 | 321 |
| 7 | k__Bacteria;p__Firmicutes;c__Clostridia;o__Clostridiales;f__Ruminococcaceae;__;__ | 386 | 276 | 598 | 565 | 527 | 912 | 1259 | 1610 | 418 | 27 | 451 | 1683 | 900 | 470 | 202 | 1642 | 322 | 170 |
| 8 | k__Bacteria;p__Firmicutes;c__Clostridia;o__Clostridiales;f__Lachnospiraceae;g__Defluviitalea;s__saccharophila | 5 | 0 | 48 | 6 | 13 | 20 | 13 | 19 | 32 | 14 | 7 | 13 | 12 | 9 | 4 | 17 | 17 | 13 |
| 9 | k__Bacteria;p__Firmicutes;c__Clostridia;o__Clostridiales;f__Lachnospiraceae;__;__ | 7234 | 3266 | 3365 | 3700 | 5012 | 3615 | 5714 | 9248 | 8940 | 5196 | 7092 | 6403 | 1355 | 5435 | 16483 | 11802 | 12416 | 5378 |
| 10 | k__Bacteria;p__Fusobacteria;c__Fusobacteriia;o__Fusobacteriales;f__Fusobacteriaceae;g__Cetobacterium;s__ | 23 | 0 | 11 | 0 | 0 | 17 | 36 | 56 | 29 | 0 | 46 | 5 | 0 | 0 | 58 | 49 | 12 | 0 |
| 11 | k__Bacteria;p__Proteobacteria;c__Deltaproteobacteria;o__Myxococcales;f__Polyangiaceae;__;__ | 0 | 4 | 0 | 0 | 3 | 0 | 4 | 3 | 0 | 0 | 0 | 0 | 0 | 0 | 0 | 0 | 0 | 0 |
| 12 | k__Bacteria;p__Firmicutes;c__Clostridia;o__Clostridiales;__;__;__ | 1287 | 3190 | 2616 | 1271 | 2026 | 1611 | 3151 | 5401 | 2283 | 1483 | 6642 | 4387 | 1991 | 1629 | 3400 | 2536 | 3600 | 2665 |
| 13 | k__Bacteria;p__Proteobacteria;c__Gammaproteobacteria;o__Xanthomonadales;f__Xanthomonadaceae;g__Thermomonas;s__dokdonensis | 2 | 1 | 8 | 8 | 13 | 1 | 0 | 9 | 16 | 17 | 18 | 22 | 17 | 13 | 15 | 21 | 0 | 0 |
| 14 | k__Bacteria;p__Firmicutes;c__Clostridia;o__Clostridiales;f__Lachnospiraceae;g__Shuttleworthia;s__satelles | 0 | 35 | 0 | 4 | 0 | 7 | 15 | 115 | 441 | 42 | 99 | 397 | 270 | 53 | 595 | 221 | 193 | 259 |
| 15 | k__Bacteria;p__Bacteroidetes;c__Bacteroidia;o__Bacteroidales;f__Rikenellaceae;g__Alistipes;__ | 2000 | 715 | 1953 | 3340 | 4580 | 1059 | 26 | 25 | 4 | 0 | 6 | 6 | 7 | 10 | 11 | 1 | 0 | 4 |
| 16 | k__Bacteria;p__Firmicutes;c__Clostridia;o__Clostridiales;f__Lachnospiraceae;g__Roseburia;s__inulinivorans | 8 | 0 | 0 | 44 | 12 | 23 | 758 | 606 | 31 | 201 | 599 | 15 | 29 | 17 | 23 | 28 | 0 | 0 |
| 17 | k__Bacteria;p__Firmicutes;c__Clostridia;o__Clostridiales;f__Lachnospiraceae;g__Dorea;s__longicatena | 146 | 55 | 142 | 79 | 73 | 292 | 4443 | 8776 | 3045 | 12318 | 7930 | 15138 | 3013 | 833 | 4502 | 1636 | 2610 | 1213 |
| 18 | k__Bacteria;p__Gemmatimonadetes;c__Gemm-5;o__;f__;g__;s__ | 12 | 8 | 7 | 4 | 6 | 5 | 5 | 14 | 0 | 0 | 0 | 0 | 0 | 0 | 0 | 0 | 0 | 0 |
| 19 | k__Bacteria;p__Firmicutes;c__Clostridia;o__Clostridiales;f__Lachnospiraceae;g__Ruminococcus;s__lactaris | 157 | 440 | 35 | 807 | 1219 | 798 | 107 | 724 | 87 | 154 | 80 | 68 | 43 | 51 | 111 | 110 | 94 | 23 |
| 20 | k__Bacteria;p__Actinobacteria;c__Actinobacteria;o__Bifidobacteriales;f__Bifidobacteriaceae;g__Bifidobacterium;__ | 0 | 0 | 0 | 0 | 0 | 0 | 0 | 12 | 52 | 23 | 0 | 0 | 0 | 0 | 0 | 0 | 0 | 0 |
| 21 | k__Bacteria;p__Proteobacteria;c__Betaproteobacteria;o__Nitrosomonadales;f__Nitrosomonadaceae;g__Nitrosomonas;__ | 18 | 34 | 15 | 17 | 63 | 7 | 541 | 205 | 187 | 1063 | 35 | 614 | 22 | 118 | 303 | 178 | 145 | 16 |
| 22 | k__Bacteria;p__Bacteroidetes;__;__;__;__;__ | 0 | 10 | 4 | 0 | 0 | 0 | 6 | 11 | 0 | 0 | 4 | 0 | 8 | 0 | 18 | 9 | 4 | 18 |
| 23 | k__Bacteria;p__Firmicutes;c__Clostridia;o__Clostridiales;f__Lachnospiraceae;g__Clostridium;__ | 3180 | 6176 | 4917 | 2253 | 2915 | 4873 | 4287 | 2329 | 2276 | 5156 | 6253 | 5019 | 1436 | 1497 | 3494 | 3582 | 4334 | 5086 |
| 24 | k__Bacteria;p__Proteobacteria;c__Gammaproteobacteria;__;__;__;__ | 9 | 4 | 2 | 5 | 0 | 3 | 0 | 3 | 11 | 6 | 0 | 9 | 0 | 0 | 0 | 4 | 5 | 0 |
| 25 | k__Bacteria;p__Chloroflexi;c__Thermomicrobia;o__JG30-KF-CM45;f__;g__;s__ | 3 | 0 | 0 | 3 | 0 | 0 | 0 | 0 | 0 | 0 | 0 | 0 | 0 | 0 | 0 | 0 | 0 | 0 |
| 26 | k__Bacteria;p__Proteobacteria;c__Gammaproteobacteria;o__Xanthomonadales;f__Xanthomonadaceae;g__Lysobacter;__ | 35 | 35 | 32 | 25 | 22 | 25 | 12 | 53 | 0 | 0 | 0 | 0 | 0 | 0 | 0 | 0 | 0 | 0 |
| 27 | k__Bacteria;p__Firmicutes;c__Erysipelotrichi;o__Erysipelotrichales;f__Erysipelotrichaceae;g__Anaerorhabdus;s__furcosa | 12 | 38 | 7 | 18 | 3 | 0 | 365 | 32 | 270 | 382 | 9 | 71 | 7 | 612 | 718 | 111 | 843 | 282 |
| 28 | k__Bacteria;p__Bacteroidetes;c__Bacteroidia;o__Bacteroidales;f__Bacteroidaceae;g__Bacteroides;s__ | 76 | 95 | 23 | 91 | 34 | 159 | 983 | 328 | 122 | 41 | 39 | 351 | 3291 | 932 | 101 | 230 | 352 | 1921 |
| 29 | k__Bacteria;p__Proteobacteria;c__Betaproteobacteria;__;__;__;__ | 668 | 531 | 513 | 682 | 689 | 475 | 24 | 148 | 229 | 21 | 9 | 193 | 1323 | 436 | 134 | 316 | 1009 | 23 |
| 30 | k__Bacteria;p__Actinobacteria;c__Coriobacteriia;o__Coriobacteriales;f__Coriobacteriaceae;__;__ | 222 | 255 | 1050 | 316 | 269 | 208 | 25 | 7 | 46 | 37 | 48 | 20 | 301 | 351 | 262 | 167 | 227 | 427 |
| 31 | k__Bacteria;p__Proteobacteria;c__Gammaproteobacteria;o__Vibrionales;f__Pseudoalteromonadaceae;__;__ | 14 | 20 | 9 | 0 | 13 | 43 | 558 | 49 | 23 | 0 | 293 | 20 | 0 | 36 | 29 | 52 | 0 | 0 |
| 32 | k__Bacteria;p__Firmicutes;c__Bacilli;o__Lactobacillales;f__Lactobacillaceae;g__Lactobacillus;__ | 5077 | 11715 | 1348 | 1780 | 7918 | 4060 | 2463 | 892 | 6994 | 2399 | 686 | 5103 | 165 | 337 | 534 | 279 | 333 | 109 |
| 33 | k__Bacteria;p__Firmicutes;c__Erysipelotrichi;o__Erysipelotrichales;f__Erysipelotrichaceae;__;__ | 15 | 0 | 6 | 0 | 3 | 0 | 0 | 3 | 5 | 0 | 3 | 3 | 22 | 35 | 61 | 0 | 22 | 16 |
| 34 | k__Bacteria;p__Proteobacteria;c__Gammaproteobacteria;o__Enterobacteriales;f__Enterobacteriaceae;__;__ | 177 | 147 | 118 | 136 | 152 | 126 | 1211 | 1667 | 4232 | 1722 | 416 | 829 | 91 | 144 | 201 | 174 | 45 | 28 |
| 35 | k__Bacteria;p__Firmicutes;c__Clostridia;o__Clostridiales;f__Peptostreptococcaceae;g__Clostridium;s__ruminantium | 17 | 9 | 25 | 13 | 12 | 5 | 140 | 205 | 19 | 456 | 65 | 604 | 70 | 349 | 1614 | 374 | 417 | 181 |
| 36 | k__Bacteria;p__Actinobacteria;c__Actinobacteria;o__Bifidobacteriales;f__Bifidobacteriaceae;g__Bifidobacterium;s__breve | 30 | 11 | 24 | 48 | 5 | 18 | 6 | 14 | 19 | 0 | 17 | 13 | 0 | 9 | 9 | 29 | 0 | 0 |
| 37 | k__Bacteria;p__Actinobacteria;c__Actinobacteria;o__Actinomycetales;f__Dietziaceae;__;__ | 0 | 0 | 0 | 0 | 0 | 0 | 0 | 0 | 8 | 0 | 11 | 0 | 0 | 0 | 10 | 0 | 0 | 0 |
| 38 | k__Bacteria;p__Proteobacteria;c__Gammaproteobacteria;o__Alteromonadales;f__OM60;g__Haliea;s__mediterranea | 0 | 0 | 6 | 4 | 4 | 0 | 0 | 7 | 0 | 0 | 0 | 0 | 0 | 0 | 0 | 0 | 0 | 0 |
| 39 | k__Bacteria;p__Firmicutes;c__Bacilli;o__Bacillales;__;__;__ | 39 | 40 | 47 | 35 | 35 | 34 | 101 | 31 | 84 | 63 | 0 | 37 | 0 | 18 | 77 | 79 | 16 | 38 |
| 40 | k__Bacteria;p__Proteobacteria;c__Gammaproteobacteria;o__Xanthomonadales;f__Xanthomonadaceae;__;__ | 23 | 7 | 10 | 9 | 2 | 9 | 0 | 10 | 16 | 8 | 6 | 11 | 0 | 0 | 11 | 15 | 5 | 0 |
| 41 | k__Bacteria;p__Firmicutes;c__Clostridia;o__Clostridiales;f__Ruminococcaceae;g__Faecalibacterium;s__ | 14 | 25 | 36 | 24 | 42 | 29 | 48 | 36 | 24 | 48 | 65 | 34 | 33 | 7 | 52 | 72 | 9 | 9 |
| 42 | k__Bacteria;p__Firmicutes;c__Clostridia;o__Clostridiales;f__Lachnospiraceae;g__Marvinbryantia;s__formatexigens | 44 | 14 | 29 | 0 | 14 | 25 | 0 | 0 | 0 | 0 | 0 | 0 | 0 | 0 | 0 | 0 | 0 | 0 |
| 43 | k__Bacteria;p__Firmicutes;c__Bacilli;o__Lactobacillales;f__Carnobacteriaceae;g__Isobaculum;s__melis | 0 | 0 | 0 | 0 | 0 | 0 | 0 | 0 | 0 | 36 | 0 | 0 | 0 | 7 | 10 | 0 | 0 | 0 |
| 44 | k__Bacteria;p__Proteobacteria;c__Betaproteobacteria;o__Burkholderiales;f__Oxalobacteraceae;g__Herbaspirillum;__ | 12 | 6 | 8 | 10 | 8 | 14 | 10 | 11 | 0 | 0 | 0 | 0 | 0 | 0 | 0 | 0 | 0 | 0 |
| 45 | k__Bacteria;p__Actinobacteria;c__Coriobacteriia;o__Coriobacteriales;f__Coriobacteriaceae;g__Paraeggerthella;s__hongkongensis | 347 | 781 | 739 | 133 | 237 | 151 | 1304 | 956 | 1152 | 2058 | 2816 | 661 | 640 | 673 | 891 | 509 | 812 | 667 |
| 46 | k__Bacteria;p__Bacteroidetes;c__Bacteroidia;o__Bacteroidales;f__Bacteroidaceae;g__Bacteroides;__ | 12 | 15 | 10 | 14 | 21 | 17 | 103 | 116 | 19 | 15 | 13 | 45 | 66 | 206 | 34 | 70 | 48 | 106 |
| 47 | k__Bacteria;p__Bacteroidetes;c__Flavobacteriia;o__Flavobacteriales;f__Flavobacteriaceae;g__Flavobacterium;s__gelidilacus | 4 | 5 | 0 | 0 | 0 | 0 | 0 | 0 | 0 | 0 | 0 | 0 | 0 | 0 | 0 | 0 | 0 | 0 |
| 48 | k__Bacteria;p__Bacteroidetes;c__Bacteroidia;o__Bacteroidales;f__;g__;s__ | 18 | 22 | 11 | 31 | 5 | 31 | 10 | 26 | 4 | 7 | 0 | 11 | 198 | 43 | 48 | 19 | 72 | 74 |
| 49 | k__Bacteria;p__Actinobacteria;c__Actinobacteria;o__Actinomycetales;f__Micrococcaceae;g__Arthrobacter;__ | 0 | 6 | 6 | 10 | 8 | 0 | 0 | 41 | 0 | 9 | 11 | 0 | 0 | 0 | 0 | 15 | 0 | 0 |
| 50 | k__Bacteria;p__Firmicutes;c__Clostridia;o__Clostridiales;f__Lachnospiraceae;g__Butyrivibrio;s__crossotus | 13 | 0 | 0 | 0 | 0 | 49 | 0 | 0 | 0 | 0 | 0 | 0 | 0 | 0 | 0 | 0 | 0 | 0 |
| 51 | k__Bacteria;p__Firmicutes;c__Erysipelotrichi;o__Erysipelotrichales;f__Erysipelotrichaceae;g__Clostridium;s__saccharogumia | 30 | 8 | 30 | 11 | 16 | 32 | 115 | 52 | 0 | 185 | 9 | 110 | 533 | 0 | 18 | 13 | 13 | 205 |
| 52 | k__Bacteria;__;__;__;__;__;__ | 237 | 189 | 513 | 349 | 291 | 388 | 221 | 439 | 320 | 49 | 46 | 221 | 64 | 325 | 319 | 295 | 262 | 170 |
| 53 | k__Bacteria;p__Bacteroidetes;c__Sphingobacteriia;o__Sphingobacteriales;f__Sphingobacteriaceae;g__Sphingobacterium;s__faecium | 2 | 0 | 0 | 0 | 2 | 4 | 0 | 10 | 0 | 0 | 0 | 0 | 0 | 0 | 0 | 0 | 0 | 0 |
| 54 | k__Bacteria;p__Firmicutes;c__Clostridia;o__Clostridiales;f__Ruminococcaceae;g__;s__ | 982 | 643 | 1593 | 686 | 1276 | 2138 | 1795 | 2783 | 164 | 67 | 432 | 992 | 781 | 3817 | 288 | 986 | 455 | 477 |
| 55 | k__Bacteria;p__Proteobacteria;c__Deltaproteobacteria;o__Desulfovibrionales;f__Desulfovibrionaceae;g__Desulfovibrio;__ | 395 | 73 | 382 | 129 | 60 | 362 | 1198 | 392 | 101 | 42 | 294 | 730 | 154 | 596 | 986 | 412 | 278 | 552 |
| 56 | k__Bacteria;p__Proteobacteria;c__Betaproteobacteria;o__Burkholderiales;f__Comamonadaceae;__;__ | 13 | 21 | 10 | 12 | 11 | 4 | 4 | 10 | 6 | 16 | 0 | 9 | 0 | 0 | 0 | 2 | 0 | 0 |
| 57 | k__Bacteria;p__Firmicutes;c__Bacilli;o__Lactobacillales;f__Lactobacillaceae;g__Lactobacillus;s__hamsteri | 7 | 11 | 0 | 10 | 0 | 20 | 25 | 27 | 26 | 83 | 13 | 12 | 0 | 10 | 37 | 22 | 0 | 0 |
| 58 | k__Bacteria;p__Bacteroidetes;c__Bacteroidia;o__Bacteroidales;f__[Barnesiellaceae];g__Barnesiella;__ | 0 | 5 | 4 | 0 | 0 | 0 | 7 | 3 | 0 | 4 | 7 | 0 | 0 | 0 | 2 | 4 | 0 | 5 |
| 59 | k__Bacteria;p__Bacteroidetes;c__[Saprospirae];o__[Saprospirales];f__Chitinophagaceae;g__Flavihumibacter;s__ | 5 | 0 | 0 | 3 | 1 | 6 | 6 | 1 | 0 | 0 | 0 | 0 | 0 | 0 | 0 | 0 | 0 | 0 |
| 60 | k__Bacteria;p__Cyanobacteria;c__Chloroplast;o__Streptophyta;f__;g__;s__ | 19 | 11 | 19 | 45 | 46 | 41 | 41 | 55 | 25 | 25 | 27 | 39 | 40 | 35 | 40 | 61 | 6 | 11 |
| 61 | k__Bacteria;p__Firmicutes;c__Clostridia;o__Clostridiales;f__Ruminococcaceae;g__Papillibacter;s__cinnamivorans | 154 | 48 | 155 | 113 | 72 | 123 | 45 | 18 | 36 | 16 | 0 | 16 | 0 | 40 | 104 | 6 | 16 | 45 |
| 62 | k__Bacteria;p__Bacteroidetes;c__Bacteroidia;o__Bacteroidales;__;__;__ | 5000 | 2851 | 2255 | 2415 | 1501 | 1962 | 1324 | 544 | 140 | 45 | 22 | 197 | 6514 | 3296 | 221 | 302 | 651 | 1146 |
| 63 | k__Bacteria;p__Proteobacteria;c__Alphaproteobacteria;o__Rhodobacterales;f__Rhodobacteraceae;__;__ | 70 | 47 | 60 | 39 | 28 | 40 | 131 | 78 | 48 | 33 | 134 | 47 | 24 | 26 | 23 | 80 | 40 | 18 |
| 64 | k__Bacteria;p__Firmicutes;c__Clostridia;o__Clostridiales;f__Peptostreptococcaceae;g__Proteocatella;s__sphenisci | 0 | 0 | 0 | 0 | 0 | 0 | 3 | 0 | 0 | 0 | 11 | 0 | 0 | 0 | 0 | 0 | 0 | 0 |
| 65 | k__Bacteria;p__Proteobacteria;c__Gammaproteobacteria;o__Xanthomonadales;f__Xanthomonadaceae;g__Lysobacter;s__oryzae | 6 | 2 | 0 | 1 | 1 | 0 | 1 | 9 | 0 | 0 | 0 | 0 | 0 | 0 | 0 | 0 | 0 | 0 |
| 66 | k__Bacteria;p__Firmicutes;c__Erysipelotrichi;o__Erysipelotrichales;f__Erysipelotrichaceae;g__Allobaculum;s__ | 2617 | 130 | 1639 | 7535 | 66 | 4417 | 107 | 563 | 3171 | 2319 | 95 | 58 | 201 | 824 | 3222 | 856 | 653 | 123 |
| 67 | k__Bacteria;p__TM7;c__TM7-3;o__CW040;f__F16;g__;s__ | 1431 | 810 | 2289 | 2491 | 913 | 2203 | 32 | 33 | 61 | 6 | 0 | 6 | 4 | 3 | 4 | 30 | 0 | 2 |
| 68 | k__Bacteria;p__Firmicutes;c__Clostridia;o__Clostridiales;f__Ruminococcaceae;g__Ruminococcus;s__ | 251 | 25 | 291 | 58 | 86 | 506 | 1567 | 376 | 485 | 965 | 163 | 327 | 100 | 351 | 543 | 218 | 979 | 324 |
| 69 | k__Bacteria;p__Proteobacteria;c__Alphaproteobacteria;o__Rhizobiales;f__Bradyrhizobiaceae;g__Nitrobacter;s__ | 0 | 0 | 0 | 0 | 0 | 0 | 0 | 0 | 9 | 0 | 0 | 0 | 7 | 6 | 0 | 17 | 0 | 0 |
| 70 | k__Bacteria;p__Firmicutes;c__Clostridia;o__Clostridiales;f__Ruminococcaceae;g__Clostridium;s__methylpentosum | 91 | 168 | 386 | 148 | 405 | 854 | 1233 | 1431 | 285 | 204 | 383 | 449 | 1828 | 1488 | 1106 | 425 | 310 | 470 |
| 71 | k__Bacteria;p__Proteobacteria;c__Gammaproteobacteria;o__Xanthomonadales;f__Xanthomonadaceae;g__Arenimonas;s__oryziterrae | 13 | 0 | 9 | 5 | 7 | 0 | 12 | 19 | 4 | 5 | 0 | 7 | 0 | 0 | 0 | 5 | 0 | 0 |
| 72 | k__Bacteria;p__Proteobacteria;c__Alphaproteobacteria;o__Rhizobiales;f__Bradyrhizobiaceae;__;__ | 0 | 0 | 0 | 0 | 0 | 0 | 8 | 0 | 0 | 21 | 12 | 17 | 0 | 0 | 14 | 0 | 0 | 0 |
| 73 | k__Bacteria;p__Gemmatimonadetes;c__Gemm-1;o__;f__;g__;s__ | 5 | 0 | 0 | 0 | 7 | 3 | 0 | 0 | 0 | 0 | 0 | 0 | 0 | 0 | 0 | 0 | 0 | 0 |
| 74 | k__Bacteria;p__Proteobacteria;c__Alphaproteobacteria;o__Rickettsiales;f__mitochondria;__;__ | 17 | 11 | 10 | 27 | 25 | 20 | 21 | 29 | 3 | 9 | 11 | 22 | 11 | 13 | 30 | 26 | 4 | 5 |
| 75 | k__Bacteria;p__Firmicutes;c__Bacilli;o__Lactobacillales;f__Streptococcaceae;g__Streptococcus;s__equi | 10 | 13 | 22 | 17 | 7 | 8 | 80 | 45 | 94 | 390 | 21 | 23 | 12 | 32 | 106 | 35 | 31 | 10 |
| 76 | k__Bacteria;p__Firmicutes;c__Bacilli;o__Bacillales;f__Bacillaceae;__;__ | 20 | 0 | 88 | 14 | 25 | 4 | 0 | 5 | 6 | 0 | 0 | 2 | 16 | 0 | 0 | 0 | 0 | 0 |
| 77 | k__Bacteria;p__Bacteroidetes;c__Bacteroidia;o__Bacteroidales;f__Prevotellaceae;g__Prevotella;s__ | 612 | 74 | 276 | 115 | 284 | 378 | 0 | 12 | 0 | 3 | 4 | 3 | 0 | 2 | 3 | 11 | 0 | 0 |
| 78 | k__Bacteria;p__Proteobacteria;c__Gammaproteobacteria;o__Pseudomonadales;f__Moraxellaceae;g__Psychrobacter;__ | 11 | 12 | 10 | 9 | 11 | 8 | 9 | 7 | 0 | 0 | 0 | 4 | 0 | 0 | 0 | 0 | 0 | 0 |
| 79 | k__Bacteria;p__Cyanobacteria;c__4C0d-2;o__YS2;f__;g__;s__ | 2 | 0 | 5 | 0 | 0 | 0 | 0 | 0 | 0 | 0 | 0 | 0 | 0 | 0 | 0 | 0 | 0 | 0 |
| 80 | k__Bacteria;p__Firmicutes;c__Clostridia;o__Clostridiales;f__Clostridiaceae;g__Clostridium;s__disporicum | 0 | 0 | 0 | 0 | 0 | 0 | 0 | 0 | 0 | 0 | 7 | 0 | 0 | 0 | 64 | 0 | 0 | 0 |
| 81 | k__Bacteria;p__Bacteroidetes;c__Bacteroidia;o__Bacteroidales;f__Bacteroidaceae;g__Bacteroides;s__acidifaciens | 406 | 455 | 186 | 676 | 813 | 867 | 93 | 125 | 35 | 11 | 7 | 66 | 422 | 422 | 242 | 264 | 445 | 305 |
| 82 | k__Bacteria;p__Bacteroidetes;c__[Saprospirae];o__[Saprospirales];f__Chitinophagaceae;__;__ | 6 | 0 | 6 | 0 | 0 | 0 | 0 | 9 | 0 | 1 | 0 | 1 | 0 | 0 | 0 | 0 | 0 | 0 |
| 83 | k__Bacteria;p__Firmicutes;__;__;__;__;__ | 47 | 10 | 218 | 88 | 116 | 40 | 546 | 334 | 4601 | 2096 | 70 | 447 | 189 | 7 | 12 | 147 | 2 | 31 |
| 84 | k__Bacteria;p__Proteobacteria;c__Betaproteobacteria;o__Burkholderiales;__;__;__ | 20 | 0 | 8 | 5 | 7 | 18 | 88 | 93 | 142 | 30 | 14 | 32 | 7 | 5 | 5 | 8 | 7 | 50 |
| 85 | k__Bacteria;p__Proteobacteria;c__Betaproteobacteria;o__Burkholderiales;f__Oxalobacteraceae;__;__ | 0 | 0 | 0 | 0 | 0 | 0 | 0 | 8 | 7 | 18 | 4 | 11 | 5 | 7 | 7 | 11 | 0 | 0 |
| 86 | k__Bacteria;p__Bacteroidetes;c__Cytophagia;o__Cytophagales;f__Flammeovirgaceae;g__Marinoscillum;__ | 10 | 0 | 0 | 0 | 0 | 0 | 4 | 6 | 0 | 0 | 0 | 0 | 0 | 0 | 0 | 0 | 0 | 0 |
| 87 | k__Bacteria;p__Firmicutes;c__Clostridia;__;__;__;__ | 0 | 2 | 17 | 0 | 0 | 4 | 0 | 0 | 0 | 0 | 0 | 0 | 0 | 12 | 0 | 11 | 2 | 0 |
| 88 | k__Bacteria;p__Firmicutes;c__Bacilli;o__Bacillales;f__Bacillaceae;g__Bacillus;s__koreensis | 0 | 0 | 0 | 0 | 0 | 0 | 0 | 0 | 5 | 9 | 0 | 0 | 0 | 4 | 0 | 0 | 0 | 0 |
| 89 | k__Bacteria;p__Proteobacteria;c__Gammaproteobacteria;o__Vibrionales;f__Vibrionaceae;g__Vibrio;s__ponticus | 0 | 0 | 0 | 0 | 0 | 0 | 8 | 0 | 0 | 0 | 9 | 0 | 0 | 0 | 0 | 0 | 0 | 0 |
| 90 | k__Bacteria;p__Actinobacteria;c__Actinobacteria;o__Actinomycetales;__;__;__ | 0 | 0 | 0 | 0 | 0 | 0 | 93 | 14 | 0 | 2 | 69 | 0 | 0 | 0 | 0 | 0 | 0 | 0 |
| 91 | k__Bacteria;p__Actinobacteria;c__Actinobacteria;o__Actinomycetales;f__Streptomycetaceae;g__Streptomyces;__ | 16 | 14 | 0 | 0 | 0 | 0 | 0 | 35 | 0 | 0 | 0 | 0 | 0 | 0 | 0 | 20 | 0 | 0 |
| 92 | k__Bacteria;p__Actinobacteria;c__Coriobacteriia;o__Coriobacteriales;f__Coriobacteriaceae;g__Adlercreutzia;s__ | 50 | 156 | 73 | 52 | 148 | 10 | 222 | 181 | 835 | 659 | 1031 | 266 | 73 | 255 | 658 | 438 | 401 | 361 |
| 93 | k__Bacteria;p__Actinobacteria;c__Actinobacteria;o__Actinomycetales;f__Microbacteriaceae;g__Leifsonia;__ | 0 | 0 | 0 | 0 | 0 | 0 | 0 | 0 | 14 | 13 | 9 | 9 | 6 | 11 | 0 | 11 | 0 | 0 |
| 94 | k__Bacteria;p__TM7;c__TM7-1;o__;f__;g__;s__ | 0 | 0 | 0 | 0 | 0 | 0 | 0 | 0 | 0 | 0 | 2 | 4 | 0 | 0 | 0 | 0 | 0 | 0 |
| 95 | k__Bacteria;p__Proteobacteria;c__Gammaproteobacteria;o__Pseudomonadales;f__Moraxellaceae;g__Acinetobacter;s__ | 30 | 36 | 36 | 42 | 49 | 28 | 33 | 45 | 26 | 54 | 20 | 26 | 23 | 16 | 21 | 17 | 7 | 5 |
| 96 | k__Bacteria;p__Firmicutes;c__Clostridia;o__Clostridiales;f__Ruminococcaceae;g__Sporobacter;s__termitidis | 141 | 24 | 70 | 344 | 40 | 251 | 26 | 64 | 19 | 0 | 0 | 10 | 0 | 6 | 0 | 60 | 0 | 0 |
| 97 | k__Bacteria;p__Firmicutes;c__Clostridia;o__Clostridiales;f__Ruminococcaceae;g__Anaerotruncus;s__ | 4 | 0 | 0 | 3 | 0 | 0 | 298 | 120 | 19 | 51 | 148 | 94 | 114 | 173 | 51 | 99 | 111 | 104 |
| 98 | k__Bacteria;p__Proteobacteria;c__Alphaproteobacteria;o__Sphingomonadales;f__Sphingomonadaceae;__;__ | 24 | 0 | 0 | 0 | 23 | 13 | 0 | 30 | 0 | 7 | 15 | 10 | 4 | 0 | 15 | 5 | 0 | 0 |
| 99 | k__Bacteria;p__Nitrospirae;c__Nitrospira;o__Nitrospirales;f__Nitrospiraceae;g__Nitrospira;s__calida | 5 | 7 | 0 | 6 | 10 | 6 | 10 | 23 | 10 | 7 | 4 | 0 | 0 | 0 | 0 | 0 | 0 | 4 |
| 100 | k__Bacteria;p__Chloroflexi;c__Gitt-GS-136;o__;f__;g__;s__ | 0 | 0 | 8 | 0 | 0 | 3 | 0 | 0 | 0 | 0 | 0 | 0 | 0 | 0 | 0 | 0 | 0 | 0 |
| 101 | k__Bacteria;p__Actinobacteria;c__Actinobacteria;o__Actinomycetales;f__Actinosynnemataceae;g__Lentzea;__ | 0 | 0 | 0 | 0 | 0 | 0 | 0 | 0 | 0 | 0 | 12 | 0 | 0 | 0 | 0 | 11 | 0 | 0 |
| 102 | k__Bacteria;p__Bacteroidetes;c__Bacteroidia;o__Bacteroidales;f__Rikenellaceae;g__Alistipes;s__massiliensis | 0 | 0 | 0 | 0 | 0 | 5 | 0 | 0 | 0 | 0 | 5 | 7 | 0 | 0 | 4 | 45 | 0 | 0 |
| 103 | k__Bacteria;p__Deferribacteres;c__Deferribacteres;o__Deferribacterales;f__Deferribacteraceae;g__Mucispirillum;s__schaedleri | 20 | 13 | 93 | 189 | 26 | 98 | 156 | 531 | 68 | 14 | 7 | 911 | 384 | 24 | 14 | 41 | 105 | 62 |
| 104 | k__Bacteria;p__Proteobacteria;c__Alphaproteobacteria;o__Rhodobacterales;f__Rhodobacteraceae;g__Shimia;s__marina | 0 | 0 | 5 | 0 | 0 | 0 | 7 | 0 | 0 | 0 | 10 | 0 | 0 | 4 | 0 | 0 | 0 | 0 |
| 105 | k__Bacteria;p__Proteobacteria;c__Gammaproteobacteria;o__Xanthomonadales;f__Xanthomonadaceae;g__Lysobacter;s__niabensis | 24 | 16 | 26 | 24 | 9 | 13 | 20 | 21 | 4 | 14 | 0 | 3 | 3 | 3 | 11 | 17 | 0 | 0 |
| 106 | k__Bacteria;p__Proteobacteria;c__Gammaproteobacteria;o__Aeromonadales;f__Aeromonadaceae;g__Aeromonas;__ | 35 | 26 | 0 | 21 | 0 | 19 | 26 | 23 | 20 | 24 | 12 | 15 | 0 | 10 | 16 | 19 | 6 | 8 |
| 107 | k__Bacteria;p__Firmicutes;c__Clostridia;o__Clostridiales;f__Clostridiaceae;g__Clostridium;__ | 0 | 0 | 0 | 0 | 0 | 4 | 6 | 10 | 6 | 0 | 0 | 0 | 4 | 2 | 163 | 9 | 0 | 0 |
| 108 | k__Bacteria;p__Firmicutes;c__Bacilli;o__Lactobacillales;__;__;__ | 0 | 0 | 0 | 0 | 0 | 0 | 0 | 12 | 0 | 11 | 8 | 0 | 4 | 0 | 0 | 4 | 0 | 0 |
| 109 | k__Bacteria;p__Firmicutes;c__Clostridia;o__Clostridiales;f__Ruminococcaceae;g__Clostridium;s__islandicum | 20 | 31 | 65 | 11 | 35 | 0 | 0 | 0 | 0 | 0 | 0 | 0 | 0 | 0 | 0 | 0 | 0 | 0 |
| 110 | k__Bacteria;p__Bacteroidetes;c__Bacteroidia;o__Bacteroidales;f__Rikenellaceae;g__Alistipes;s__finegoldii | 186 | 45 | 149 | 109 | 190 | 186 | 68 | 58 | 95 | 0 | 0 | 0 | 0 | 138 | 112 | 79 | 199 | 109 |
| 111 | k__Bacteria;p__Firmicutes;c__Bacilli;o__Lactobacillales;f__Lactobacillaceae;g__Lactobacillus;s__mucosae | 4 | 4 | 5 | 0 | 7 | 6 | 3 | 6 | 0 | 0 | 0 | 0 | 0 | 0 | 8 | 2 | 0 | 0 |
| 112 | k__Bacteria;p__Firmicutes;c__Bacilli;o__Bacillales;f__Staphylococcaceae;g__Macrococcus;__ | 0 | 0 | 0 | 0 | 0 | 0 | 9 | 0 | 5 | 3 | 3 | 0 | 2 | 0 | 10 | 0 | 0 | 0 |
| 113 | k__Bacteria;p__Bacteroidetes;c__Bacteroidia;o__Bacteroidales;f__Rikenellaceae;g__AF12;s__ | 187 | 148 | 208 | 165 | 135 | 203 | 18 | 138 | 59 | 21 | 16 | 192 | 306 | 175 | 189 | 93 | 602 | 568 |
| 114 | k__Bacteria;p__Verrucomicrobia;c__Verrucomicrobiae;o__Verrucomicrobiales;f__Verrucomicrobiaceae;g__Akkermansia;s__muciniphila | 28 | 39 | 717 | 40 | 19 | 34 | 1029 | 330 | 106 | 2969 | 246 | 34 | 23167 | 86 | 40 | 95 | 124 | 25954 |
| 115 | k__Bacteria;p__Proteobacteria;c__Epsilonproteobacteria;o__Campylobacterales;f__Helicobacteraceae;__;__ | 264 | 418 | 867 | 591 | 949 | 445 | 781 | 1501 | 110 | 115 | 32 | 787 | 898 | 59 | 11 | 72 | 198 | 377 |
| 116 | k__Bacteria;p__Proteobacteria;c__Gammaproteobacteria;o__Xanthomonadales;f__Xanthomonadaceae;g__Aspromonas;s__composti | 0 | 0 | 0 | 0 | 0 | 0 | 0 | 0 | 0 | 0 | 1 | 0 | 0 | 0 | 3 | 1 | 0 | 0 |
| 117 | k__Bacteria;p__Firmicutes;c__Clostridia;o__Clostridiales;f__Peptostreptococcaceae;g__[Clostridium];s__litorale | 0 | 0 | 0 | 0 | 0 | 0 | 0 | 7 | 3 | 0 | 0 | 15 | 0 | 0 | 3 | 9 | 0 | 6 |
| 118 | k__Bacteria;p__Firmicutes;c__Clostridia;o__Clostridiales;f__Clostridiaceae;g__Clostridium;s__purinilyticum | 17 | 8 | 31 | 0 | 6 | 0 | 0 | 0 | 0 | 0 | 0 | 0 | 0 | 0 | 0 | 0 | 0 | 0 |
| 119 | k__Bacteria;p__Proteobacteria;c__Gammaproteobacteria;o__Vibrionales;f__Pseudoalteromonadaceae;g__Pseudoalteromonas;s__luteoviolacea | 10 | 9 | 10 | 7 | 11 | 0 | 63 | 16 | 10 | 4 | 47 | 6 | 3 | 3 | 3 | 9 | 0 | 0 |
| 120 | k__Bacteria;p__Firmicutes;c__Clostridia;o__Clostridiales;f__Ruminococcaceae;g__Clostridium;__ | 34 | 287 | 113 | 0 | 122 | 129 | 21 | 12 | 77 | 17 | 3 | 8 | 0 | 3 | 0 | 20 | 0 | 0 |
| 121 | k__Bacteria;p__Firmicutes;c__Bacilli;o__Lactobacillales;f__Lactobacillaceae;g__Lactobacillus;s__ | 0 | 0 | 0 | 0 | 0 | 0 | 0 | 0 | 8 | 0 | 10 | 0 | 0 | 0 | 0 | 0 | 0 | 0 |
| 122 | k__Bacteria;p__Bacteroidetes;c__Bacteroidia;o__Bacteroidales;f__Porphyromonadaceae;g__Macellibacteroides;s__fermentans | 104 | 157 | 50 | 140 | 82 | 333 | 184 | 663 | 15 | 12 | 9 | 97 | 1465 | 1477 | 294 | 203 | 510 | 250 |
| 123 | k__Bacteria;p__Firmicutes;c__Clostridia;o__Clostridiales;f__Peptostreptococcaceae;g__[Clostridium];s__difficile | 0 | 0 | 0 | 0 | 0 | 0 | 0 | 6 | 5 | 0 | 4 | 0 | 0 | 0 | 0 | 7 | 0 | 0 |
| 124 | k__Bacteria;p__Proteobacteria;c__Alphaproteobacteria;o__Rhizobiales;__;__;__ | 6 | 8 | 0 | 0 | 4 | 0 | 4 | 3 | 0 | 0 | 0 | 0 | 0 | 0 | 0 | 0 | 0 | 0 |
| 125 | k__Bacteria;p__Acidobacteria;c__[Chloracidobacteria];o__RB41;f__Ellin6075;g__;s__ | 0 | 0 | 0 | 0 | 0 | 0 | 0 | 0 | 3 | 0 | 0 | 0 | 3 | 0 | 0 | 0 | 0 | 0 |
| 126 | k__Bacteria;p__Firmicutes;c__Bacilli;__;__;__;__ | 18 | 26 | 9 | 4 | 35 | 0 | 3 | 0 | 11 | 5 | 0 | 8 | 0 | 0 | 0 | 0 | 0 | 0 |
| 127 | k__Bacteria;p__Chloroflexi;c__Anaerolineae;o__SBR1031;f__SHA-31;g__;s__ | 0 | 0 | 0 | 0 | 0 | 0 | 0 | 0 | 2 | 2 | 0 | 0 | 0 | 0 | 0 | 0 | 0 | 0 |
| 128 | k__Bacteria;p__Proteobacteria;c__Deltaproteobacteria;o__Myxococcales;f__Haliangiaceae;g__;s__ | 0 | 0 | 0 | 0 | 0 | 3 | 0 | 4 | 2 | 0 | 0 | 0 | 0 | 0 | 0 | 4 | 0 | 0 |
| 129 | k__Bacteria;p__Proteobacteria;c__Alphaproteobacteria;o__Sphingomonadales;f__Sphingomonadaceae;g__Sphingosinicella;s__microcystinivorans | 16 | 0 | 17 | 13 | 12 | 12 | 15 | 16 | 15 | 39 | 14 | 18 | 10 | 0 | 17 | 18 | 0 | 0 |
| 130 | k__Bacteria;p__Cyanobacteria;__;__;__;__;__ | 0 | 0 | 0 | 0 | 0 | 0 | 718 | 0 | 5 | 3 | 0 | 0 | 0 | 0 | 2 | 2 | 0 | 0 |
| 131 | k__Bacteria;p__Actinobacteria;c__Actinobacteria;o__Bifidobacteriales;f__Bifidobacteriaceae;g__Alloscardovia;s__ | 0 | 0 | 0 | 0 | 0 | 0 | 8 | 10 | 37 | 119 | 12 | 0 | 0 | 16 | 16 | 5 | 0 | 0 |
| 132 | k__Bacteria;p__Proteobacteria;c__Betaproteobacteria;o__Burkholderiales;f__Oxalobacteraceae;g__Massilia;__ | 20 | 19 | 12 | 21 | 15 | 10 | 15 | 27 | 0 | 5 | 2 | 11 | 8 | 10 | 0 | 15 | 0 | 0 |
| 133 | k__Bacteria;p__Firmicutes;c__Clostridia;o__Clostridiales;f__Lachnospiraceae;g__Clostridium;s__fimetarium | 0 | 0 | 188 | 0 | 9 | 0 | 0 | 0 | 0 | 0 | 0 | 0 | 0 | 0 | 0 | 0 | 0 | 0 |
| 134 | k__Bacteria;p__Bacteroidetes;c__[Saprospirae];o__[Saprospirales];f__Chitinophagaceae;g__Trachelomonas;s__volvocinopsis | 4 | 0 | 0 | 6 | 8 | 4 | 4 | 10 | 0 | 0 | 0 | 0 | 0 | 0 | 0 | 0 | 0 | 0 |
| 135 | k__Bacteria;p__Bacteroidetes;c__Cytophagia;o__Cytophagales;f__Cytophagaceae;g__;s__ | 2 | 4 | 0 | 8 | 10 | 0 | 8 | 13 | 0 | 0 | 0 | 0 | 0 | 0 | 0 | 2 | 0 | 0 |
| 136 | k__Bacteria;p__Firmicutes;c__Bacilli;o__Bacillales;f__Planococcaceae;g__Staphylococcus;s__saprophyticus | 8 | 0 | 0 | 8 | 0 | 13 | 178 | 24 | 45 | 35 | 0 | 20 | 14 | 11 | 19 | 32 | 22 | 9 |
| 137 | k__Bacteria;p__Proteobacteria;c__Deltaproteobacteria;o__Desulfovibrionales;__;__;__ | 40 | 11 | 12 | 41 | 0 | 111 | 838 | 429 | 283 | 182 | 239 | 343 | 163 | 258 | 237 | 490 | 537 | 362 |
| 138 | k__Bacteria;p__Proteobacteria;c__Betaproteobacteria;o__Methylophilales;f__Methylophilaceae;g__Methylotenera;s__versatilis | 0 | 4 | 0 | 0 | 0 | 0 | 0 | 5 | 0 | 0 | 0 | 0 | 0 | 0 | 0 | 0 | 0 | 0 |
| 139 | k__Bacteria;p__Proteobacteria;c__Alphaproteobacteria;o__Rhodobacterales;f__Hyphomonadaceae;g__Hellea;s__balneolensis | 0 | 0 | 0 | 0 | 0 | 0 | 17 | 0 | 0 | 0 | 10 | 0 | 0 | 0 | 0 | 0 | 0 | 0 |
| 140 | k__Bacteria;p__Proteobacteria;c__Gammaproteobacteria;o__Pseudomonadales;f__Pseudomonadaceae;__;__ | 0 | 0 | 0 | 0 | 0 | 0 | 32 | 0 | 0 | 0 | 22 | 0 | 0 | 0 | 0 | 0 | 0 | 0 |
| 141 | k__Bacteria;p__Actinobacteria;c__Acidimicrobiia;o__Acidimicrobiales;f__C111;g__Ilumatobacter;s__fluminis | 0 | 10 | 0 | 0 | 0 | 0 | 0 | 36 | 0 | 0 | 0 | 0 | 4 | 0 | 0 | 16 | 0 | 0 |
| 142 | k__Bacteria;p__GAL15;c__;o__;f__;g__;s__ | 0 | 0 | 0 | 0 | 0 | 0 | 0 | 226 | 0 | 0 | 0 | 0 | 0 | 0 | 0 | 47 | 0 | 0 |
| 143 | k__Bacteria;p__Proteobacteria;c__Alphaproteobacteria;o__Rhodospirillales;f__Rhodospirillaceae;g__Skermanella;s__xinjiangensis | 3 | 0 | 0 | 0 | 3 | 0 | 0 | 0 | 0 | 0 | 15 | 0 | 0 | 0 | 0 | 0 | 0 | 0 |
| 144 | k__Bacteria;p__Firmicutes;c__Clostridia;o__Clostridiales;f__Veillonellaceae;__;__ | 0 | 0 | 0 | 0 | 0 | 6 | 0 | 0 | 4 | 0 | 0 | 5 | 0 | 0 | 0 | 13 | 0 | 0 |
| 145 | k__Bacteria;p__Firmicutes;c__Clostridia;o__Clostridiales;f__Ruminococcaceae;g__Oscillospira;s__guilliermondii | 84 | 20 | 175 | 432 | 254 | 250 | 65 | 50 | 0 | 6 | 113 | 49 | 11 | 67 | 15 | 244 | 25 | 135 |
| 146 | k__Bacteria;p__WS3;c__PRR-12;o__Sediment-1;f__PRR-10;g__;s__ | 0 | 0 | 0 | 0 | 0 | 0 | 0 | 0 | 0 | 0 | 3 | 0 | 4 | 0 | 3 | 0 | 0 | 0 |
| 147 | k__Bacteria;p__Actinobacteria;c__Acidimicrobiia;o__Acidimicrobiales;f__SC3-41;g__;s__ | 0 | 0 | 0 | 0 | 0 | 0 | 0 | 0 | 0 | 0 | 20 | 0 | 0 | 3 | 0 | 0 | 0 | 0 |
| 148 | k__Bacteria;p__Firmicutes;c__Clostridia;o__Clostridiales;f__Lachnospiraceae;g__Clostridium;s__symbiosum | 33 | 62 | 62 | 0 | 0 | 0 | 2502 | 40 | 51 | 847 | 223 | 104 | 0 | 59 | 1634 | 34 | 21 | 28 |
| 149 | k__Bacteria;p__Proteobacteria;c__Deltaproteobacteria;o__Desulfovibrionales;f__Desulfovibrionaceae;g__Desulfovibrio;s__C21_c20 | 288 | 5454 | 4834 | 1438 | 3224 | 181 | 1211 | 550 | 85 | 62 | 731 | 243 | 689 | 789 | 37 | 461 | 159 | 1785 |
| 150 | k__Bacteria;p__Proteobacteria;c__Betaproteobacteria;o__Burkholderiales;f__Alcaligenaceae;g__Bordetella;s__ansorpii | 7 | 4 | 0 | 1 | 0 | 4 | 2 | 0 | 0 | 0 | 0 | 0 | 0 | 0 | 0 | 0 | 0 | 0 |
| 151 | k__Bacteria;p__Proteobacteria;c__Betaproteobacteria;o__Burkholderiales;f__Comamonadaceae;g__Aquamonas;s__fontana | 1 | 0 | 0 | 0 | 0 | 1 | 0 | 0 | 0 | 0 | 0 | 0 | 0 | 0 | 0 | 0 | 0 | 0 |
| 152 | k__Bacteria;p__Proteobacteria;c__Gammaproteobacteria;o__Oceanospirillales;f__Saccharospirillaceae;__;__ | 0 | 0 | 0 | 0 | 0 | 0 | 25 | 0 | 0 | 0 | 24 | 0 | 0 | 0 | 0 | 0 | 0 | 0 |
| 153 | k__Bacteria;p__Firmicutes;c__Bacilli;o__Lactobacillales;f__Enterococcaceae;g__Enterococcus;s__cecorum | 0 | 0 | 0 | 0 | 0 | 0 | 4 | 0 | 0 | 0 | 0 | 0 | 0 | 0 | 7 | 12 | 0 | 0 |
| 154 | k__Bacteria;p__Gemmatimonadetes;c__;o__;f__;g__;s__ | 0 | 0 | 0 | 0 | 0 | 4 | 0 | 0 | 0 | 0 | 0 | 0 | 0 | 0 | 0 | 3 | 0 | 0 |
| 155 | k__Bacteria;p__Bacteroidetes;c__Bacteroidia;o__Bacteroidales;f__Porphyromonadaceae;g__Parabacteroides;s__gordonii | 14 | 15 | 11 | 13 | 30 | 13 | 22 | 25 | 0 | 0 | 5 | 19 | 168 | 28 | 19 | 29 | 16 | 29 |
| 156 | k__Bacteria;p__Bacteroidetes;c__Flavobacteriia;o__Flavobacteriales;f__Flavobacteriaceae;g__Actibacter;s__sediminis | 0 | 0 | 4 | 0 | 5 | 0 | 0 | 0 | 0 | 0 | 2 | 2 | 5 | 0 | 0 | 3 | 0 | 0 |
| 157 | k__Bacteria;p__Proteobacteria;c__Deltaproteobacteria;o__Desulfovibrionales;f__Desulfovibrionaceae;__;__ | 2653 | 346 | 937 | 2277 | 1135 | 4130 | 10163 | 30843 | 5974 | 4840 | 4371 | 15503 | 15952 | 12313 | 14201 | 32060 | 24536 | 13887 |
| 158 | k__Bacteria;p__Actinobacteria;c__Coriobacteriia;o__Coriobacteriales;f__Coriobacteriaceae;g__Olsenella;s__profusa | 90 | 22 | 112 | 104 | 28 | 49 | 215 | 2735 | 20309 | 15392 | 4084 | 66 | 74 | 1089 | 847 | 1253 | 2380 | 6 |
| 159 | k__Bacteria;p__Proteobacteria;c__Alphaproteobacteria;o__Rhizobiales;f__Brucellaceae;g__Pseudochrobactrum;s__ | 11 | 10 | 6 | 0 | 0 | 0 | 0 | 0 | 0 | 0 | 0 | 0 | 0 | 0 | 0 | 0 | 0 | 0 |
| 160 | k__Bacteria;p__Firmicutes;c__Bacilli;o__Lactobacillales;f__Leuconostocaceae;g__Weissella;__ | 4 | 5 | 9 | 3 | 0 | 4 | 4 | 0 | 0 | 0 | 0 | 2 | 0 | 0 | 0 | 0 | 7 | 3 |
| 161 | k__Bacteria;p__Firmicutes;c__Clostridia;o__Clostridiales;f__Lachnospiraceae;g__Clostridium;s__aldenense | 112 | 268 | 45 | 22 | 42 | 79 | 0 | 0 | 0 | 0 | 0 | 0 | 0 | 0 | 0 | 0 | 0 | 0 |
| 162 | k__Bacteria;p__Actinobacteria;__;__;__;__;__ | 19 | 0 | 0 | 6 | 11 | 0 | 0 | 20 | 0 | 0 | 0 | 0 | 0 | 0 | 0 | 0 | 0 | 0 |
| 163 | k__Bacteria;p__Proteobacteria;c__Epsilonproteobacteria;o__Campylobacterales;f__Helicobacteraceae;g__Helicobacter;s__hepaticus | 22 | 31 | 83 | 95 | 187 | 110 | 6 | 47 | 12 | 0 | 0 | 16 | 6 | 7 | 0 | 12 | 0 | 0 |
| 164 | k__Bacteria;p__Cyanobacteria;c__Chloroplast;__;__;__;__ | 0 | 0 | 0 | 0 | 0 | 0 | 11 | 0 | 0 | 0 | 2 | 0 | 0 | 0 | 0 | 0 | 0 | 0 |
| 165 | k__Bacteria;p__Proteobacteria;c__Alphaproteobacteria;o__Rhodobacterales;__;__;__ | 0 | 0 | 0 | 0 | 0 | 0 | 8 | 0 | 0 | 0 | 8 | 0 | 0 | 0 | 0 | 0 | 0 | 0 |
| 166 | k__Bacteria;p__Proteobacteria;c__Alphaproteobacteria;o__Sphingomonadales;f__Erythrobacteraceae;__;__ | 0 | 0 | 0 | 0 | 0 | 0 | 19 | 0 | 0 | 0 | 9 | 0 | 0 | 0 | 0 | 0 | 0 | 0 |
| 167 | k__Bacteria;p__Proteobacteria;c__Gammaproteobacteria;o__Thiotrichales;f__Thiotrichaceae;g__Allobeggiatoa;s__halophila | 9 | 4 | 0 | 0 | 0 | 0 | 0 | 0 | 3 | 4 | 0 | 0 | 0 | 0 | 0 | 0 | 3 | 3 |
| 168 | k__Bacteria;p__Bacteroidetes;c__Flavobacteriia;o__Flavobacteriales;f__[Weeksellaceae];g__Wautersiella;s__ | 0 | 5 | 3 | 0 | 0 | 0 | 4 | 4 | 0 | 0 | 0 | 0 | 0 | 2 | 2 | 0 | 0 | 0 |
| 169 | k__Bacteria;p__Tenericutes;c__Mollicutes;o__Entomoplasmatales;f__Entomoplasmataceae;g__Entomoplasma;s__somnilux | 0 | 0 | 0 | 0 | 0 | 0 | 0 | 0 | 6 | 5 | 6 | 6 | 0 | 6 | 5 | 12 | 0 | 0 |
| 170 | k__Bacteria;p__Firmicutes;c__Erysipelotrichi;o__Erysipelotrichales;f__Erysipelotrichaceae;g__Clostridium;__ | 15 | 4 | 8 | 0 | 19 | 0 | 479 | 263 | 188 | 916 | 7 | 199 | 1107 | 550 | 1047 | 422 | 177 | 3670 |
| 171 | k__Bacteria;p__Actinobacteria;c__Actinobacteria;o__Actinomycetales;f__Corynebacteriaceae;g__Corynebacterium;s__pilosum | 0 | 0 | 0 | 0 | 0 | 0 | 10 | 5 | 9 | 8 | 15 | 0 | 0 | 0 | 0 | 0 | 0 | 0 |
| 172 | k__Bacteria;p__Proteobacteria;c__Gammaproteobacteria;o__Vibrionales;__;__;__ | 0 | 9 | 22 | 8 | 12 | 0 | 36 | 0 | 0 | 28 | 53 | 0 | 17 | 0 | 0 | 0 | 0 | 10 |
| 173 | k__Bacteria;p__Firmicutes;c__Clostridia;o__Clostridiales;f__Christensenellaceae;g__Christensenella;s__ | 27 | 0 | 21 | 7 | 11 | 0 | 38 | 8 | 12 | 25 | 6 | 4 | 53 | 104 | 3 | 11 | 48 | 32 |
| 174 | k__Bacteria;p__Bacteroidetes;c__Flavobacteriia;o__Flavobacteriales;f__Flavobacteriaceae;g__Coccinimonas;s__marina | 0 | 0 | 0 | 3 | 4 | 0 | 0 | 0 | 0 | 0 | 0 | 0 | 0 | 0 | 0 | 0 | 0 | 0 |
| 175 | k__Bacteria;p__Actinobacteria;c__Acidimicrobiia;o__Acidimicrobiales;f__koll13;g__mixed;s__culture isolate koll13 | 6 | 5 | 0 | 0 | 0 | 0 | 0 | 0 | 6 | 0 | 0 | 0 | 0 | 0 | 0 | 0 | 6 | 0 |
| 176 | k__Bacteria;p__Firmicutes;c__Bacilli;o__Lactobacillales;f__Streptococcaceae;__;__ | 0 | 3 | 0 | 0 | 0 | 3 | 54 | 14 | 43 | 215 | 5 | 17 | 5 | 22 | 88 | 12 | 17 | 0 |
| 177 | k__Bacteria;p__Verrucomicrobia;c__Verrucomicrobiae;o__Verrucomicrobiales;f__Verrucomicrobiaceae;g__Haloferula;s__rosea | 10 | 4 | 6 | 2 | 5 | 5 | 0 | 6 | 6 | 0 | 0 | 0 | 0 | 0 | 0 | 7 | 0 | 0 |
| 178 | k__Bacteria;p__Verrucomicrobia;c__Verrucomicrobiae;o__Verrucomicrobiales;f__Verrucomicrobiaceae;g__Haloferula;__ | 0 | 0 | 0 | 0 | 1 | 3 | 3 | 5 | 5 | 9 | 0 | 11 | 3 | 3 | 3 | 6 | 0 | 0 |
| 179 | k__Bacteria;p__Verrucomicrobia;c__Verrucomicrobiae;o__Verrucomicrobiales;f__Verrucomicrobiaceae;g__Persicirhabdus;s__sediminis | 0 | 0 | 0 | 0 | 0 | 0 | 0 | 3 | 0 | 0 | 10 | 0 | 0 | 0 | 0 | 0 | 0 | 0 |
| 180 | k__Bacteria;p__Actinobacteria;c__Acidimicrobiia;o__Acidimicrobiales;f__Iamiaceae;g__;s__ | 5 | 0 | 0 | 0 | 6 | 5 | 0 | 0 | 0 | 0 | 0 | 0 | 0 | 0 | 0 | 0 | 0 | 0 |
| 181 | k__Bacteria;p__Firmicutes;c__Bacilli;o__Lactobacillales;f__Lactobacillaceae;g__Lactobacillus;s__pontis | 25 | 0 | 0 | 0 | 0 | 0 | 0 | 24 | 895 | 0 | 39 | 0 | 0 | 0 | 0 | 0 | 0 | 0 |
| 182 | k__Bacteria;p__Proteobacteria;c__Alphaproteobacteria;o__Sphingomonadales;f__Sphingomonadaceae;g__Sphingomonas;__ | 0 | 23 | 22 | 0 | 0 | 0 | 21 | 0 | 0 | 0 | 0 | 0 | 0 | 0 | 0 | 0 | 0 | 0 |
| 183 | k__Bacteria;p__Actinobacteria;c__Coriobacteriia;o__Coriobacteriales;f__Coriobacteriaceae;g__Enterococcus;s__casseliflavus | 0 | 4 | 6 | 0 | 4 | 0 | 0 | 0 | 0 | 0 | 0 | 0 | 0 | 0 | 0 | 0 | 0 | 0 |
| 184 | k__Bacteria;p__Firmicutes;c__Bacilli;o__Lactobacillales;f__Leuconostocaceae;g__Leuconostoc;s__mesenteroides | 0 | 0 | 0 | 0 | 0 | 0 | 7 | 0 | 27 | 109 | 0 | 7 | 0 | 10 | 20 | 0 | 10 | 0 |
| 185 | k__Bacteria;p__Proteobacteria;c__Gammaproteobacteria;o__Alteromonadales;f__Alteromonadaceae;g__Saccharophagus;s__degradans | 3 | 0 | 6 | 0 | 0 | 0 | 0 | 6 | 0 | 0 | 0 | 0 | 0 | 0 | 0 | 0 | 0 | 0 |
| 186 | k__Bacteria;p__Verrucomicrobia;c__Verrucomicrobiae;o__Verrucomicrobiales;f__Verrucomicrobiaceae;g__Haloferula;s__harenae | 10 | 11 | 8 | 10 | 9 | 8 | 12 | 15 | 0 | 0 | 0 | 0 | 0 | 0 | 0 | 0 | 0 | 0 |
| 187 | k__Bacteria;p__Proteobacteria;c__Alphaproteobacteria;o__Rhizobiales;f__Brucellaceae;g__Ochrobactrum;__ | 0 | 0 | 0 | 7 | 0 | 0 | 7 | 9 | 8 | 14 | 20 | 0 | 0 | 0 | 8 | 15 | 0 | 0 |
| 188 | k__Bacteria;p__Proteobacteria;c__Gammaproteobacteria;o__Oceanospirillales;__;__;__ | 0 | 0 | 3 | 5 | 0 | 0 | 0 | 5 | 0 | 0 | 0 | 0 | 0 | 0 | 0 | 2 | 0 | 0 |
| 189 | k__Bacteria;p__Proteobacteria;c__Deltaproteobacteria;o__Desulfovibrionales;f__Desulfovibrionaceae;g__Bilophila;s__ | 0 | 0 | 0 | 3 | 0 | 0 | 0 | 5 | 0 | 0 | 0 | 0 | 0 | 0 | 0 | 6 | 0 | 0 |
| 190 | k__Bacteria;p__Firmicutes;c__Clostridia;o__Clostridiales;f__Lachnospiraceae;g__Clostridium;s__colinum | 5 | 187 | 368 | 0 | 33 | 18 | 0 | 3 | 0 | 0 | 0 | 0 | 0 | 0 | 0 | 0 | 0 | 0 |
| 191 | k__Bacteria;p__Proteobacteria;c__Alphaproteobacteria;o__Rhodobacterales;f__Rhodobacteraceae;g__Marinovum;s__algicola | 0 | 0 | 0 | 0 | 0 | 0 | 12 | 0 | 0 | 0 | 21 | 0 | 0 | 0 | 0 | 18 | 0 | 0 |
| 192 | k__Bacteria;p__Firmicutes;c__Clostridia;o__Clostridiales;f__Ruminococcaceae;g__Gemmiger;s__formicilis | 0 | 0 | 0 | 0 | 0 | 0 | 0 | 6 | 0 | 0 | 0 | 0 | 0 | 5 | 5 | 0 | 0 | 2 |
| 193 | k__Bacteria;p__Bacteroidetes;c__Bacteroidia;o__Bacteroidales;f__Rikenellaceae;g__Alistipes;s__indistinctus | 25 | 69 | 25 | 27 | 26 | 22 | 0 | 0 | 0 | 0 | 0 | 0 | 16 | 32 | 33 | 47 | 35 | 3 |
| 194 | k__Bacteria;p__;c__;o__;f__;g__;s__ | 4 | 4 | 0 | 2 | 6 | 0 | 0 | 0 | 0 | 0 | 0 | 0 | 0 | 0 | 0 | 0 | 0 | 0 |
| 195 | k__Bacteria;p__Tenericutes;c__CK-1C4-19;o__;f__;g__;s__ | 0 | 0 | 0 | 0 | 0 | 0 | 0 | 8 | 0 | 0 | 0 | 0 | 0 | 0 | 0 | 13 | 0 | 0 |
| 196 | k__Bacteria;p__Chlamydiae;c__Chlamydiia;o__Chlamydiales;f__Rhabdochlamydiaceae;g__Candidatus Rhabdochlamydia;s__ | 0 | 0 | 0 | 0 | 0 | 0 | 9 | 0 | 0 | 0 | 0 | 0 | 0 | 0 | 10 | 0 | 0 | 0 |
| 197 | k__Bacteria;p__Proteobacteria;c__Gammaproteobacteria;o__Vibrionales;f__Pseudoalteromonadaceae;g__Vibrio;s__mimicus | 19 | 0 | 0 | 0 | 0 | 0 | 0 | 0 | 12 | 0 | 0 | 0 | 0 | 0 | 0 | 0 | 0 | 0 |
| 198 | k__Bacteria;p__Proteobacteria;c__Gammaproteobacteria;o__Pseudomonadales;f__Pseudomonadaceae;g__Pseudomonas;__ | 0 | 0 | 0 | 0 | 9 | 6 | 14 | 0 | 0 | 12 | 8 | 7 | 0 | 0 | 0 | 12 | 5 | 0 |
| 199 | k__Bacteria;p__Proteobacteria;c__Alphaproteobacteria;o__Rhodobacterales;f__Rhodobacteraceae;g__Sulfitobacter;__ | 0 | 0 | 0 | 0 | 0 | 0 | 33 | 0 | 0 | 0 | 20 | 0 | 0 | 0 | 0 | 0 | 0 | 0 |
| 200 | k__Bacteria;p__Proteobacteria;c__Alphaproteobacteria;o__Rhodobacterales;f__Rhodobacteraceae;g__Thalassobius;s__gelatinovorus | 0 | 0 | 0 | 0 | 0 | 0 | 41 | 0 | 0 | 0 | 15 | 0 | 0 | 0 | 0 | 0 | 0 | 0 |
| 201 | k__Bacteria;p__Bacteroidetes;c__[Saprospirae];o__[Saprospirales];f__Chitinophagaceae;g__Parasegitibacter;s__luojiensis | 8 | 5 | 0 | 0 | 0 | 0 | 0 | 11 | 0 | 2 | 0 | 2 | 0 | 3 | 0 | 7 | 0 | 0 |
| 202 | k__Bacteria;p__Proteobacteria;c__Gammaproteobacteria;o__Chromatiales;__;__;__ | 17 | 14 | 0 | 0 | 0 | 0 | 0 | 0 | 14 | 12 | 0 | 0 | 0 | 0 | 0 | 0 | 4 | 5 |
| 203 | k__Bacteria;p__Bacteroidetes;c__Bacteroidia;o__Bacteroidales;f__[Paraprevotellaceae];g__[Prevotella];s__ | 24 | 11 | 6 | 10 | 3 | 5 | 0 | 0 | 0 | 0 | 0 | 0 | 7 | 8 | 0 | 2 | 0 | 0 |
| 204 | k__Bacteria;p__Chloroflexi;c__Anaerolineae;o__SBR1031;f__A4b;g__;s__ | 0 | 2 | 0 | 0 | 0 | 0 | 0 | 3 | 0 | 0 | 0 | 0 | 0 | 0 | 0 | 0 | 0 | 0 |
| 205 | k__Bacteria;p__Proteobacteria;c__Alphaproteobacteria;__;__;__;__ | 0 | 0 | 10 | 17 | 0 | 0 | 0 | 0 | 0 | 0 | 0 | 0 | 0 | 0 | 0 | 0 | 0 | 0 |
| 206 | k__Bacteria;p__Armatimonadetes;c__0319-6E2;o__;f__;g__;s__ | 0 | 0 | 0 | 6 | 0 | 0 | 0 | 4 | 0 | 0 | 0 | 0 | 3 | 0 | 0 | 0 | 0 | 0 |
| 207 | k__Bacteria;p__Proteobacteria;c__Alphaproteobacteria;o__Rhodospirillales;f__Acetobacteraceae;g__Acidisphaera;s__rubrifaciens | 0 | 0 | 0 | 0 | 0 | 0 | 0 | 0 | 0 | 8 | 0 | 0 | 2 | 0 | 5 | 13 | 0 | 0 |
| 208 | k__Bacteria;p__Firmicutes;c__Clostridia;o__Clostridiales;f__Ruminococcaceae;g__Ruminococcus;s__bromii | 0 | 4 | 0 | 0 | 0 | 4 | 0 | 0 | 0 | 0 | 0 | 0 | 0 | 0 | 0 | 0 | 0 | 0 |
| 209 | k__Bacteria;p__Firmicutes;c__Bacilli;o__Lactobacillales;f__Aerococcaceae;g__Atopostipes;s__suicloacalis | 0 | 0 | 0 | 0 | 0 | 0 | 11 | 7 | 7 | 13 | 6 | 14 | 0 | 0 | 0 | 0 | 0 | 0 |
| 210 | k__Bacteria;p__Planctomycetes;c__[Brocadiae];o__Brocadiales;f__Brocadiaceae;g__Kuenenia;s__stuttgartiensis | 5 | 2 | 0 | 0 | 0 | 0 | 0 | 0 | 5 | 2 | 0 | 0 | 0 | 0 | 0 | 0 | 0 | 0 |
| 211 | k__Bacteria;p__Proteobacteria;c__Alphaproteobacteria;o__Sphingomonadales;f__Erythrobacteraceae;g__Porphyrobacter;__ | 0 | 16 | 17 | 17 | 0 | 16 | 0 | 14 | 0 | 0 | 0 | 0 | 0 | 0 | 0 | 0 | 0 | 0 |
| 212 | k__Bacteria;p__Proteobacteria;c__Alphaproteobacteria;o__Rhodobacterales;f__Rhodobacteraceae;g__Paracoccus;s__kawasakiensis | 0 | 0 | 0 | 0 | 0 | 0 | 0 | 0 | 0 | 0 | 7 | 0 | 0 | 0 | 8 | 0 | 0 | 0 |
| 213 | k__Bacteria;p__Planctomycetes;c__Planctomycetia;o__Gemmatales;f__Isosphaeraceae;g__Nostocoida;s__limicola III | 0 | 0 | 0 | 0 | 0 | 0 | 0 | 0 | 0 | 0 | 13 | 0 | 0 | 0 | 6 | 0 | 0 | 0 |
| 214 | k__Bacteria;p__Proteobacteria;c__Gammaproteobacteria;o__Enterobacteriales;f__Enterobacteriaceae;g__Proteus;s__vulgaris | 0 | 0 | 0 | 0 | 0 | 0 | 0 | 17 | 0 | 0 | 0 | 0 | 0 | 5 | 0 | 17 | 0 | 0 |
| 215 | k__Bacteria;p__Actinobacteria;c__Actinobacteria;o__Actinomycetales;f__Brevibacteriaceae;g__Brevibacterium;s__casei | 0 | 0 | 0 | 0 | 0 | 0 | 0 | 0 | 3 | 0 | 0 | 0 | 0 | 0 | 0 | 5 | 0 | 0 |
| 216 | k__Bacteria;p__Cyanobacteria;c__Chloroplast;o__Stramenopiles;f__;g__;s__ | 0 | 0 | 0 | 0 | 0 | 0 | 95 | 0 | 0 | 0 | 5 | 0 | 0 | 0 | 0 | 0 | 0 | 0 |
| 217 | k__Bacteria;p__Bacteroidetes;c__Bacteroidia;o__Bacteroidales;f__[Paraprevotellaceae];g__Paraprevotella;s__ | 3 | 5 | 6 | 0 | 5 | 0 | 0 | 0 | 0 | 0 | 0 | 0 | 0 | 0 | 0 | 0 | 0 | 0 |
| 218 | k__Bacteria;p__Firmicutes;c__Bacilli;o__Lactobacillales;f__Lactobacillaceae;g__Lactobacillus;s__paraplantarum | 0 | 0 | 0 | 0 | 0 | 0 | 0 | 6 | 0 | 0 | 0 | 0 | 0 | 0 | 9 | 0 | 0 | 0 |
| 219 | k__Bacteria;p__Bacteroidetes;c__Bacteroidia;o__Bacteroidales;f__[Odoribacteraceae];g__Odoribacter;s__ | 0 | 0 | 3 | 0 | 0 | 0 | 4 | 8 | 0 | 0 | 0 | 0 | 0 | 0 | 2 | 0 | 0 | 0 |
| 220 | k__Bacteria;p__Actinobacteria;c__Acidimicrobiia;o__Acidimicrobiales;f__OCS155;g__;s__ | 0 | 0 | 0 | 0 | 0 | 0 | 4 | 0 | 0 | 0 | 12 | 0 | 0 | 0 | 0 | 0 | 0 | 0 |
| 221 | k__Bacteria;p__Bacteroidetes;c__Flavobacteriia;o__Flavobacteriales;__;__;__ | 0 | 0 | 9 | 4 | 0 | 4 | 5 | 6 | 0 | 6 | 10 | 3 | 0 | 4 | 0 | 0 | 0 | 0 |
| 222 | k__Bacteria;p__Proteobacteria;c__Epsilonproteobacteria;o__Campylobacterales;f__Helicobacteraceae;g__Helicobacter;s__ganmani | 9 | 0 | 26 | 29 | 75 | 16 | 14 | 6 | 754 | 12 | 8 | 9 | 0 | 56 | 637 | 119 | 204 | 4 |
| 223 | k__Bacteria;p__Bacteroidetes;c__Cytophagia;o__Cytophagales;f__Flammeovirgaceae;g__Reichenbachiella;s__ | 0 | 0 | 0 | 0 | 0 | 0 | 15 | 0 | 0 | 0 | 5 | 0 | 0 | 0 | 0 | 0 | 0 | 0 |
| 224 | k__Bacteria;p__Proteobacteria;c__Gammaproteobacteria;o__Oceanospirillales;f__Halomonadaceae;__;__ | 0 | 5 | 0 | 0 | 0 | 0 | 0 | 0 | 0 | 5 | 0 | 0 | 0 | 0 | 0 | 0 | 0 | 0 |
| 225 | k__Bacteria;p__Proteobacteria;__;__;__;__;__ | 0 | 0 | 1 | 0 | 2 | 0 | 0 | 0 | 0 | 0 | 0 | 0 | 0 | 0 | 0 | 0 | 0 | 0 |
| 226 | k__Bacteria;p__Proteobacteria;c__Gammaproteobacteria;o__Vibrionales;f__Pseudoalteromonadaceae;g__Aliivibrio;s__fischeri | 14 | 1 | 0 | 0 | 0 | 7 | 0 | 0 | 0 | 0 | 0 | 0 | 0 | 6 | 0 | 5 | 0 | 0 |
| 227 | k__Bacteria;p__Chloroflexi;c__Anaerolineae;o__H39;f__;g__;s__ | 0 | 0 | 0 | 0 | 0 | 0 | 0 | 0 | 0 | 2 | 0 | 0 | 0 | 0 | 4 | 5 | 0 | 0 |
| 228 | k__Bacteria;p__Bacteroidetes;c__Sphingobacteriia;o__Sphingobacteriales;f__Sphingobacteriaceae;__;__ | 2 | 0 | 0 | 0 | 4 | 0 | 0 | 0 | 0 | 0 | 0 | 0 | 4 | 0 | 0 | 0 | 0 | 0 |
| 229 | k__Bacteria;p__Proteobacteria;c__Gammaproteobacteria;o__Alteromonadales;f__Alteromonadaceae;__;__ | 0 | 0 | 0 | 0 | 0 | 0 | 67 | 0 | 0 | 0 | 22 | 0 | 0 | 0 | 0 | 0 | 0 | 0 |
| 230 | k__Bacteria;p__Proteobacteria;c__Alphaproteobacteria;o__RF32;f__;g__;s__ | 0 | 0 | 0 | 0 | 0 | 0 | 0 | 9 | 0 | 0 | 4 | 6 | 13 | 5 | 0 | 0 | 0 | 0 |
| 231 | k__Bacteria;p__Bacteroidetes;c__Sphingobacteriia;o__Sphingobacteriales;f__Sphingobacteriaceae;g__Sphingobacterium;s__multivorum | 0 | 0 | 0 | 2 | 0 | 0 | 3 | 0 | 0 | 0 | 0 | 0 | 0 | 0 | 0 | 0 | 0 | 0 |
| 232 | k__Bacteria;p__Actinobacteria;c__Actinobacteria;o__Actinomycetales;f__Nocardioidaceae;__;__ | 0 | 0 | 0 | 0 | 0 | 0 | 0 | 69 | 0 | 0 | 0 | 0 | 0 | 0 | 0 | 43 | 0 | 0 |
| 233 | k__Bacteria;p__Firmicutes;c__Bacilli;o__Bacillales;f__Planococcaceae;__;__ | 0 | 0 | 0 | 0 | 0 | 0 | 10 | 7 | 0 | 0 | 0 | 9 | 0 | 0 | 26 | 0 | 0 | 0 |
| 234 | k__Bacteria;p__Proteobacteria;c__Gammaproteobacteria;o__Vibrionales;f__Vibrionaceae;g__Vibrio;__ | 0 | 0 | 0 | 0 | 0 | 0 | 67 | 0 | 0 | 0 | 18 | 0 | 0 | 0 | 0 | 0 | 0 | 0 |
| 235 | k__Bacteria;p__Cyanobacteria;c__Chloroplast;o__UA01;f__;g__;s__ | 0 | 0 | 0 | 0 | 0 | 0 | 68 | 7 | 0 | 0 | 7 | 0 | 0 | 0 | 0 | 0 | 0 | 0 |
| 236 | k__Bacteria;p__TM7;c__TM7-3;o__EW055;f__;g__;s__ | 0 | 0 | 0 | 0 | 0 | 0 | 0 | 0 | 4 | 0 | 4 | 4 | 0 | 0 | 6 | 0 | 0 | 0 |
| 237 | k__Bacteria;p__Bacteroidetes;c__Flavobacteriia;o__Flavobacteriales;f__Flavobacteriaceae;g__Aquimarina;s__brevivitae | 0 | 0 | 0 | 0 | 0 | 0 | 29 | 0 | 0 | 0 | 4 | 0 | 0 | 0 | 0 | 0 | 0 | 0 |
| 238 | k__Bacteria;p__Firmicutes;c__Clostridia;o__Clostridiales;f__Ruminococcaceae;g__Oscillospira;s__ | 3 | 0 | 0 | 0 | 0 | 5 | 0 | 0 | 0 | 0 | 0 | 0 | 0 | 0 | 0 | 0 | 0 | 0 |

**Table S4 Changes in pathway abundance between the three groups**

| **Pathway increased by HFD from CD but reversed by QP treatment** | |
| --- | --- |
| Metacyc | Description |
| PWY-6210 | 2-aminophenol→egradation |
| PWY0-42 | 2-methylcitrate cycle I |
| PWY-5747 | 2-methylcitrate cycle II |
| PWY-3781 | aerobic respiration I (cytochrome c) |
| PWY0-41 | allantoin degradation IV (anaerobic) |
| PWY-5705 | allantoin degradation to glyoxylate III |
| ARGORNPROST-PWY | arginine, ornithine and proline interconversion |
| GLUCARDEG-PWY | D-glucarate degradation I |
| ECASYN-PWY | enterobacterial common antigen biosynthesis |
| ENTBACSYN-PWY | enterobactin biosynthesis |
| PWY-7094 | fatty acid salvage |
| PWY-1861 | formaldehyde assimilation II (RuMP Cycle) |
| RUMP-PWY | formaldehyde oxidation I |
| GLYOXYLATE-BYPASS | glyoxylate cycle |
| HEME-BIOSYNTHESIS-II | heme biosynthesis I (aerobic) |
| KETOGLUCONMET-PWY | ketogluconate metabolism |
| AST-PWY | L-arginine degradation II (AST pathway) |
| PWY-5430 | meta cleavage pathway of aromatic compounds |
| PWY0-1533 | methylphosphonate degradation I |
| NAD-BIOSYNTHESIS-II | NAD salvage pathway II |
| P221-PWY | octane oxidation |
| PWY0-1338 | polymyxin resistance |
| PPGPPMET-PWY | ppGpp biosynthesis |
| P23-PWY | reductive TCA cycle I |
| SUCSYN-PWY | sucrose biosynthesis I (from photosynthesis) |
| PWY-7446 | sulfoglycolysis |
| PWY-5918 | superpathay of heme biosynthesis from glutamate |
| KDO-NAGLIPASYN-PWY | superpathway of (Kdo)2-lipid A biosynthesis |
| ALL-CHORISMATE-PWY | superpathway of chorismate metabolism |
| PWY-5860 | superpathway of demethylmenaquinol-6 biosynthesis I |
| PWY-5862 | superpathway of demethylmenaquinol-9 biosynthesis |
| GLYCOL-GLYOXDEG-PWY | superpathway of glycol metabolism and degradation |
| GLYCOLYSIS-TCA-GLYOX-BYPASS | superpathway of glycolysis, pyruvate dehydrogenase, TCA, and glyoxylate bypass |
| TCA-GLYOX-BYPASS | superpathway of glyoxylate bypass and TCA |
| PWY0-1415 | superpathway of heme biosynthesis from uroporphyrinogen-III |
| HEXITOLDEGSUPER-PWY | superpathway of hexitol degradation (bacteria) |
| LPSSYN-PWY | superpathway of lipopolysaccharide biosynthesis |
| PWY-6629 | superpathway of L-tryptophan biosynthesis |
| PWY-5896 | superpathway of menaquinol-10 biosynthesis |
| PWY-5850 | superpathway of menaquinol-6 biosynthesis I |
| PWY-5845 | superpathway of menaquinol-9 biosynthesis |
| METHGLYUT-PWY | superpathway of methylglyoxal degradation |
| ORNDEG-PWY | superpathway of ornithine degradation |
| PWY-6338 | superpathway of vanillin and vanillate degradation |
| PWY-7097 | vanillin and vanillate degradation I |
| PWY-7098 | vanillin and vanillate degradation II |
|  |  |
| Pathway decreased by HFD from CD but reversed by QP treatment | |
| PWY-6121 | 5-aminoimidazole ribonucleotide biosynthesis I |
| RIBOSYN2-PWY | flavin biosynthesis I (bacteria and plants) |
| PWY-5659 | GDP-mannose biosynthesis |
| PWY-7221 | guanosine ribonucleotides de novo biosynthesis |
| PWY-6123 | inosine-5'-phosphate biosynthesis I |
| PANTOSYN-PWY | pantothenate and coenzyme A biosynthesis I |
| POLYISOPRENSYN-PWY | polyisoprenoid biosynthesis (E. coli) |
| PWY-7323 | superpathway of GDP-mannose-derived O-antigen building blocks biosynthesis |
| PWY-5695 | urate biosynthesis/inosine 5'-phosphate degradation |

**Table S5 Superclass ontologies of Metacyc pathways altered by HFD and by HFD+QP**

| Pathway increased by HFD from CD but reversed by QP treatment | | |
| --- | --- | --- |
| Metacyc | | Superclass ontologies |
| PWY-6210 | | DUA→ Aromatic Compound Degradation |
| PWY0-42 | | DUA→ Carboxylic Acid Degradation →Propanoate Degradation → 2-Methylcitrate Cycle |
| PWY-5747 | | DUA → Carboxylic Acid Degradation → Propanoate Degradation→ 2-Methylcitrate Cycle |
| PWY-3781 | | Generation of Precursor Metabolites and Energy → Electron Transfer Chains |
| PWY0-41 | | DUA → Amide, Amidine, Amine, and Polyamine Degradation → Allantoin Degradation |
| PWY-5705 | | DUA → Amide, Amidine, Amine, and Polyamine Degradation → Allantoin Degradation |
| ARGORNPROST-PWY | | DUA → Amino Acid Degradation→ Proteinogenic Amino Acid Degradation → L-arginine Degradation |
| GLUCARDEG-PWY | | DUA → Carboxylic Acid Degradation → Aldaric Acid Degradation → D-Glucarate Degradation |
| ECASYN-PWY | | Biosynthesis → CB → Polysaccharide Biosynthesis → Glycan Pathways → Polysaccharide Biosynthesis |
| ENTBACSYN-PWY | | Biosynthesis → Secondary Metabolite Biosynthesis → Siderophore and Metallophore Biosynthesis |
| PWY-7094 | | Biosynthesis → Fatty Acid and Lipid Biosynthesis → Fatty Acid Biosynthesis |
| PWY-1861 | | DUA → C1 Compound Utilization and Assimilation → Formaldehyde Assimilation |
| RUMP-PWY | | DUA → C1 Compound Utilization and Assimilation → Formaldehyde Oxidation |
| GLYOXYLATE-BYPASS | | Generation of Precursor Metabolites and Energy |
| HEME-BIOSYNTHESIS-II | | Biosynthesis → CCVB → Enzyme Cofactor Biosynthesis → Heme b Biosynthesis |
| KETOGLUCONMET-PWY | | DUA → Carboxylic Acid Degradation → Aldonic Acid Degradation |
| AST-PWY | | DUA → Amino Acid Degradation → Proteinogenic Amino Acid Degradation → L-arginine Degradation |
| PWY-5430 | | DUA → Aromatic Compound Degradation → Benzoate Degradation |
| PWY0-1533 | | DUA → Inorganic Nutrient Metabolism → Phosphonate Compound Degradation → Methylphosphonate Degradation |
| NAD-BIOSYNTHESIS-II | | Biosynthesis → CCVB → Carrier Biosynthesis → ECB → NAD Metabolism → NAD Biosynthesis |
| P221-PWY | | DUA → DUA - Other |
| PWY0-1338 | | Biosynthesis → CSB → LPS Biosynthesis Detoxification → Antibiotic Resistance Glycan Pathways → LPS Biosynthesis |
| PPGPPMET-PWY | | Biosynthesis → Metabolic Regulator Biosynthesis |
| P23-PWY | | DUA → C1 Compound Utilization and Assimilation → CO2 Fixation → Autotrophic CO2 Fixation → Reductive TCA Cycles |
| SUCSYN-PWY | | Biosynthesis → CB → Sugar Biosynthesis → Disaccharide Biosynthesis → Sucrose Biosynthesis |
| PWY-7446 | | DUA → Carbohydrate Degradation → Sulfoquinovose Degradation |
| PWY-5918 | | Biosynthesis → CCVB → Enzyme Cofactor Biosynthesis → Heme Biosynthesis → Heme b Biosynthesis |
| KDO-NAGLIPASYN-PWY | | Biosynthesis → CSB → LPS Biosynthesis → Fatty Acid and Lipid Biosynthesis Glycan Pathways → LPS Biosynthesis |
| ALL-CHORISMATE-PWY | | Biosynthesis amino acids trp,tyr, phe, THF4, menaquinol-8 enterobactin |
| PWY-5860 | | Biosynthesis → CCVB → Carrier Biosynthesis → ECB → QQB → DM-menaquinol Biosynthesis → DM-menaquinol-6 Biosynthesis |
| PWY-5862 | | Biosynthesis → CCVB → Carrier Biosynthesis → ECB → QQB → DM-menaquinol Biosynthesis |
| GLYCOL-GLYOXDEG-PWY | | DUA → Alcohol Degradation |
| GLYCOLYSIS-TCA-GLYOX-BYPASS | | Generation of Precursor Metabolites and Energy |
| TCA-GLYOX-BYPASS | | Generation of energy, TCA cycle, glyoxalate shunt (avoids loss CO2 |
| PWY0-1415 | | Biosynthesis → CCVB → Enzyme Cofactor Biosynthesis → Heme Biosynthesis → Heme b Biosynthesis |
| HEXITOLDEGSUPER-PWY | | DUA → Alcohol Degradation → Alditol Degradation DUA → Carbohydrate Degradation → Alditol Degradation |
| LPSSYN-PWY | | Biosynthesis → Fatty Acid and Lipid Biosynthesis |
| PWY-6629 | | Biosynthesis → Amino Acid Biosynthesis → Proteinogenic Amino Acid Biosynthesis → L-tryptophan Biosynthesis |
| PWY-5896 | | Biosynthesis → CCVB → Carrier Biosynthesis → ECB → QQB → Menaquinol Biosynthesis |
| PWY-5850 | | Biosynthesis → CCVB → Carrier Biosynthesis → ECB → QQB → Menaquinol Biosynthesis |
| PWY-5845 | | Biosynthesis → CCVB → Carrier Biosynthesis → ECB → QQB → Menaquinol Biosynthesis |
| METHGLYUT-PWY | | DUA → Aldehyde Degradation |
| ORNDEG-PWY | | DUA → Amide, Amidine, Amine, and Polyamine Degradation |
| PWY-6338 | | DUA → Aromatic Compound Degradation → Vanillin Degradation |
| PWY-7097 | | DUA → Aromatic Compound Degradation → Vanillin Degradation |
| PWY-7098 | | DUA → Aromatic Compound Degradation → Vanillin Degradation |
|  | |  |
| Pathway decreased by HFD from CD but reversed by QP treatment | | |
| PWY-6121 | | Biosynthesis → NNB → PNB → 5-Aminoimidazole Ribonucleotide Biosynthesis |
| RIBOSYN2-PWY | | Biosynthesis → CCVB → Carrier Biosynthesis → ECB → Flavin Biosynthesis |
| PWY-5659 | | Biosynthesis → CB → Sugar Biosynthesis → Sugar Nucleotide Biosynthesis → GDP-sugar Biosynthesis |
| PWY-7221 | | Biosynthesis → NNB → PNB → PNDNB → Purine Riboucleotide De Novo Biosynthesis |
| PWY-6123 | | Biosynthesis → NNB → PNB → PNDNB → Purine Riboucleotide De Novo Biosynthesis → Inosine-5'-phosphate Biosynthesis |
| PANTOSYN-PWY | | Biosynthesis → CCVB → Carrier Biosynthesis → Coenzyme A Biosynthesis |
| POLYISOPRENSYN-PWY | | Biosynthesis → Polyprenyl Biosynthesis |
| PWY-7323 | | Biosynthesis → CB → Sugar Biosynthesis → Sugar Nucleotide Biosynthesis → GDP-sugar Biosynthesis |
| PWY-5695 | | DUA → Nucleoside and Nucleotide Degradation → Purine Nucleotide Degradation |
|  | |  |
|  | DUA, Degradation/Utilization/Assimilation; CCVB, Cofactor, Carrier, and Vitamin Biosynthesis; | |
|  | LPS, Lipopolysaccharide; ECB, Electron Carrier Biosynthesis; NNB, Nucleoside and Nucleotide Biosynthesis; PNB, Purine Nucleotide Biosynthesis; PNDNB, Purine Nucleotide De Novo Biosynthesis; QQB, Quinol and Quinone Biosynthesis; CB, Carbohydrate Biosynthesis; CSB, Cell Structure Biosynthesis | |
|  |  | |
|  |  | |
|  |  | |
|  |  | |
